# Determinants of effective lentivirus-driven microRNA expression *in vivo*

**DOI:** 10.1038/srep33345

**Published:** 2016-09-15

**Authors:** Takuya Mishima, Elena Sadovsky, Margaret E. Gegick, Yoel Sadovsky

**Affiliations:** 1 Magee-Womens Research Institute, Department of Obstetrics, Gynaecology and Reproductive Sciences, University of Pittsburgh, Pittsburgh, Pennsylvania, 15213 USA

## Abstract

Manipulation of microRNA (miRNA) levels, including overexpression of mature species, has become an important biological tool, even motivating miRNA-based therapeutics. To assess key determinants of miRNA overexpression in a mammalian system *in vivo*, we sought to bypass the laborious generation of a transgenic animal by exploiting placental trophoblast-specific gene manipulation using lentiviral vectors, which has been instrumental in elucidating trophoblast biology. We examined the impact of several key components of miRNA stem loops and their flanking sequences on the efficiency of mature miRNA expression *in vivo.* By combining established and novel approaches for miRNA expression, we engineered lentivirus-driven miRNA expression plasmids, which we tested in the mouse placenta. We found that reverse sense inserts minimized single-strand splicing and degradation, and that maintaining longer, poly-A-containing arms flanking the miRNA stem-loop markedly enhanced transgenic miRNA expression. Additionally, we accomplished overexpression of diverse mammalian, *drosophila*, or *C. elegans* miRNAs, either based on native context or using a “cassette” replacement of the mature miRNA sequence. Together, we have identified primary miRNA sequences that are paramount for effective expression of mature miRNAs, and validated their role in mice. Principles established by our findings may guide the design of efficient miRNA vectors for *in vivo* use.

MicroRNAs (miRNAs) are 19- to 24-nt small non-coding RNAs that are processed from primary miRNA (pri-miRNA) through intermediate stem-loop precursor miRNA (pre-miRNA) structures^1^,^2^,^3^. While some miRNAs are species- or organ-specific, others are ubiquitous and conserved throughout evolution. Examples include the ubiquitously expressed mir-107^4^, the liver-predominant mir-122^5^, the ubiquitously expressed, *drosophila*-specific mir-14, mir-276a, and mir-2b^6^, the ubiquitously expressed, *C. elegans*-specific mir-77 and mir-230^1^,^7^, and the primate-specific, placenta-predominant miRNA from the Chromosome19 miRNA cluster (C19MC)^8^,^9^. Mature miRNAs regulate gene expression at the mRNA level to degrade mRNA, inhibit RNA translation, regulate genomic DNA methylation, and even regulate the expression of other miRNAs^10^,^11^,^12^. MiRNAs play roles in numerous biological processes, such as development, cancer, metabolism, and even reprogramming^10^,^13^,^14^,^15^.

Seeking to define their biological functions, researchers have used diverse approaches to optimize the design of miRNA expression vectors. Early miRNA vectors were constructed to express short-hairpin RNAs (shRNAs) that included a miRNA loop and U6 terminator, under the control of RNA polymerase III promoter^16^. Diverse shRNA species were cloned into a miRNA “cassette” construct that included the desired shRNA, with flanking sequences from highly expressed miRNA sequences such as mir-155 and mir-30^17^,^18^. Subsequent strategies included the insertion of an shRNA as an intron within marker mRNA, such as enhanced green fluorescent protein (EGFP)^17^,^18^. Recently, Li *et al.* established an eGFP-intron splicing system, where the intron (such as shRNA) is located inside the EGFP coding site^19^; thus, the presence of the EGFP signal guarantees the splicing of the intronic shRNA. These intron-splicing systems are effective when used in conjunction with double-stranded plasmid DNA or the DNA of adeno-associated virus. In contrast, the lentiviral genome comprises single-stranded RNA (ssRNA). Therefore, when the expression cassette is located in the positive-sense orientation, the shRNA intron might be a target for splicing and degradation after being transcribed from the plasmid DNA and prior to integration into the host genomic DNA. A solution recently proposed by Cooper *et al.* was based upon cloning of the lentiviral intron in a negative-sense orientation^20^, thus evading splicing prior to genomic integration.

We sought to assess key determinants of lentiviral-driven miRNA expression in the placenta *in vivo*. In eutherian organisms, the placenta is critical for embryonic development and growth and for maternal-foetal adaptation to pregnancy^21^,^22^,^23^. Placental trophoblasts, which are bathed in maternal blood in human and murine pregnancy, support pregnancy by regulating the exchange of gas, nutrients, and waste between the mother and foetus, producing hormones that are essential for pregnancy and providing immunological defence to the allogeneic foetus. Several groups have recently introduced an efficient method for murine placenta-specific gene expression^24^,^25^,^26^, in which lentivirus vectors are essential due to their potent transduction efficiency in the blastocyst’s trophectoderm layer. This system has been recently deployed to address trophoblast gene function and placental pathophysiology^27^,^28^,^29^,^30^. Building on recent advances in the field, as well as our findings described here, we designed a novel lentivirus-based miRNA expression system for *in vivo* use. We tested this system by transfecting mouse placentas with diverse types of miRNAs. As expected, we found that inverted intronic miRNAs are expressed at a much higher level than forward inserts. We also showed that intron size had a major effect on splicing efficiency *in vivo*. Importantly, we showed that members of the poly(A)-binding protein (PABP) family are germane for lentivirus-expressed miRNA prior to genomic integration.

## Results

### Trophoblast lineage-specific gene manipulation

**Figure 1:**
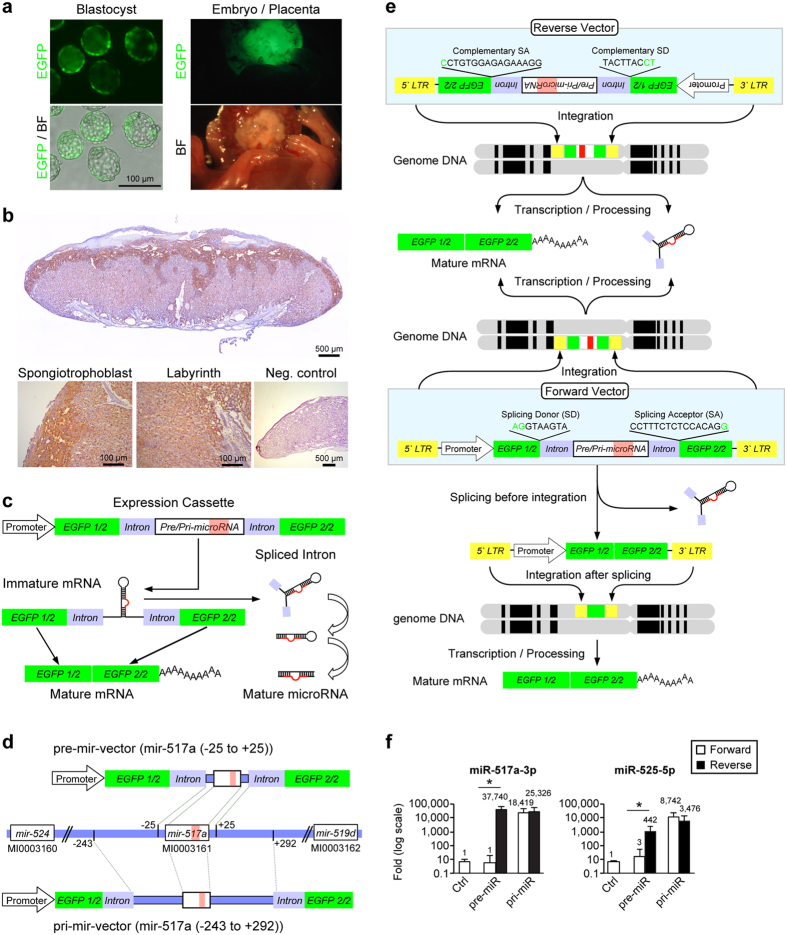
Trophoblast lineage-specific gene manipulation and expression strategy. (**a**) Blastocysts after lentivirus exposure under 488 filter set (upper left), and a combined image with bright field (BF) (bottom left). Embryo and placenta after abdominal delivery on E18.5 under 488 filter set (upper right) and under bright field (BF) (bottom right). (**b**) The expression of EGFP protein in the mouse placenta, detected by immunohistochemistry as described in Methods. Low (2X) and high (10X) magnifications show GFP expression in the labyrinth and junctional zone. (**c**) A schematic depiction of the eGFP-intron splicing system, designed to express mature miRNA. (**d**) A schematic depiction of pre-mir-vector and pri-mir-vector of mir-517a. (**e**) A schematic depiction of the forward- and reverse-vectors, showing the significance of pre-integration splicing in our design strategy. (**f**) RT-qPCR analysis of expression of miR-517a-3p (upper graph, n = 7–10) and miR-525-5p (lower graph, n = 5–10) for mouse placentas, performed separately for each vector. *Denotes p < 0.01.

The pregnancy rate and other experimental parameters are shown in Fig. S1a. In each procedure, the control blastocysts were incubated in a CO_2_ incubator for 3 days after exposure to each lentiviral construct and EGFP signal was observed exclusively in the trophectoderm of all blastocysts between 24 and 72 h (Fig. 1a, left). After transabdominal delivery at E18.5, all the placentas displayed EGFP expression under fluorescence dissecting microscope. Further confirmationby immunohistochemistry, revealed expression in the labyrinthine and junctional zones (Fig. 1a, right, and Fig. 1b). For all tested placentas, the mRNAs for EGFP (Fig. S1b) and for the integrated lentivirus were detected using RT-qPCR (Fig. S1c).

### The expression of lentivirally transduced, primate-specific C19MC-derived miRNAs

We initially attempted to overexpress miR-517a-3p and miR-525-5p in the mouse placenta using four different lentivector-based strategies. Our general intron-splicing approach is shown in Fig. 1b. The pre-mir vector has the shorter genomic DNA fragment, including the stem-loop structure with 25-nt flanks (Fig. 1c), and the pri-mir-vector has the longer, 200- to 300-nt flanks (Fig. 1c). We also deployed the reverse vector strategy described by Cooper *et al.*^20^, designed to prevent pre-integration intron splicing (as detailed in Methods). When we deployed the pri-mir vector strategy, we noted a similar level of high miRNA expression, irrespective of the orientation of the expression cassette. In contrast, when we deployed the pre-mir vector strategy, we detected a marked difference, with high miRNA expression using reverse pre-mir-vectors and very low expression using the forward pre-mir vectors (Fig. 1e).

### Overexpression of non-mammalian *(drosophila, C. elegans)* and endogenous mouse miRNAs

**Figure 2:**
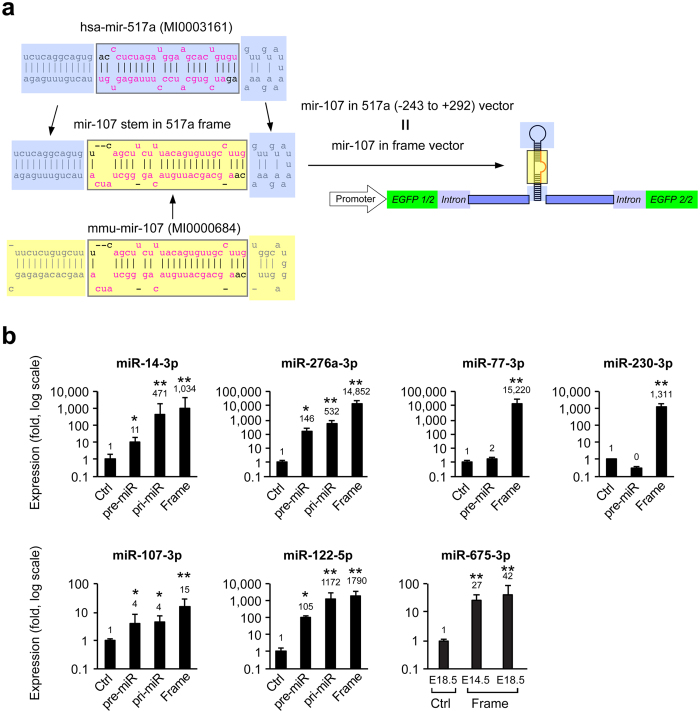
The use of “Cassette” design for the overexpression of non-mammalian miRNAs in the mouse placenta. (**a**) A schematic depiction of cassette vectors, shown for the mouse miR-107 (see further details in Methods). (**b**) The expression level of different miRNAs, determined by RT-qPCR. This included the *drosophila* miR-14 (n = 5–9) and miR-276a (n = 5–15), the *C. elegans* miR-77 (n = 5–12) and miR-230 (n = 6–10), and mouse miR-107-3p (n = 6–15), miR-122-5p (n = 3–12), and miR-675-3p (n = 6–11). *Denotes p < 0.05, **denotes p < 0.01.

It was previously reported that miRNAs derived from *C. elegans* or other lower species are difficult to express efficiently in mammalian cells *in vitro*^1^. We therefore sought to compare the expression efficiency of endogenous mouse miRNAs (mir-107, mir-122, mir-675) and exogenous miRNAs from two non-mammalian species, namely *drosophila* (mir-14 and mir-276), and *C. elegans* (mir-77, mir-230). To maximize expression, we combined our reverse pri-mir vector strategy with a “cassette” design, exploiting the high expression we had attained for mammalian miR-517a-3p. Thus, we replaced the sequence of the mature miR-517-5p and miR-517a-3p, located within the stem region within the reverse-pri-mir-517a vector (called “frame vector”), with each of the mouse endogenous and exogenous miRNAs (Fig. 2a), and assessed their expression level following transduction of the mouse blastocysts (Fig. 2b). We were able to recapitulate the lower expression level of *C. elegans* (miR-77-3p and miR-230-3p) miRNA and, to a lesser extent, of *drosophila* miRNA (miR-14-3p and miR-276a-3p), using reverse pri-mir or pre-mir strategy. Strikingly, we observed markedly enhanced expression using the “cassette” vector strategy (Fig. 2b). This approach also led to the effective expression of endogenous mouse miRNAs, including miR-107-3p, miR-122-5p, and miR-675-3p (Fig. 2b).

### The effect of intron length on miRNA expression levels

**Figure 3:**
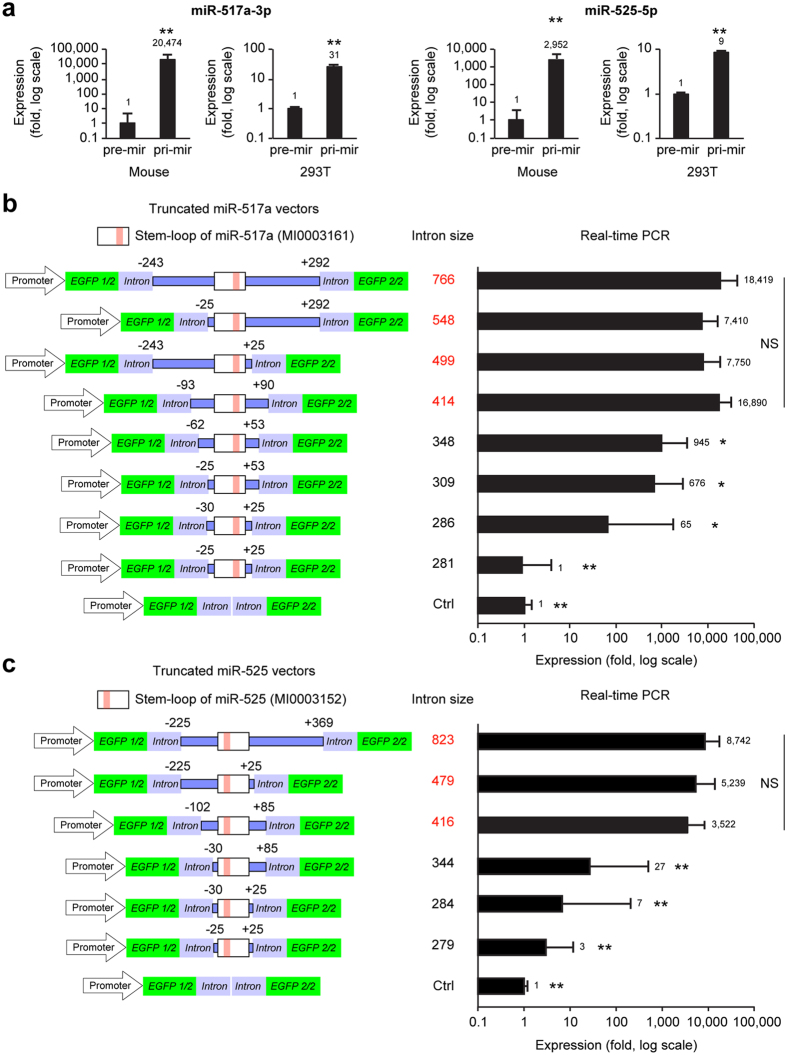
The effect of intron size on miRNA expression efficiency. (**a**) The expression levels of miR-517a-3p and miR-525-5p using forward-pre-mir-vector and forward-pri-mir-vector in the mouse placenta *in vivo* (n = 5–12) and in 293T cells *in vitro* (n = 3). (**b**,**c**) A truncation series of the flanking arms within the mir-517a vector (**b**, n = 5–20) or the mir-525 vector (**c**, n = 5–21). The left panel schematics depict the truncation series, and the right panels depict expression levels determined using RT-qPCR. *Denotes p < 0.05, **denotes p < 0.01.

As shown in Fig. 1e, we observed a marked difference in placental expression of miR-517a-3p and miR-525-5p. This was dependent upon whether a forward-pre-mir-vector or a forward-pri-mir-vector was used. We observed similar differences using the same lentivirus transduction protocol in 293T cells *in vitro*, albeit of a smaller magnitude (Fig. 3a). We therefore surmised that intron length might affect cellular splicing efficiency. To assess this possibility, we created a series of stepwise truncations in the flanking arms of either the mir-517a (Fig. 3b) or the mir-525 vector (Fig. 3c) and expressed these constructs in the mouse placenta as described earlier. As shown in Fig. 3b,c, we found that a total intron size of >400 bp resulted in the highest expression, with a gradual reduction in expression levels when we used shorter flanking arms.

### The effect of the PABP binding site of mir-517a on splicing efficiency

**Figure 4:**
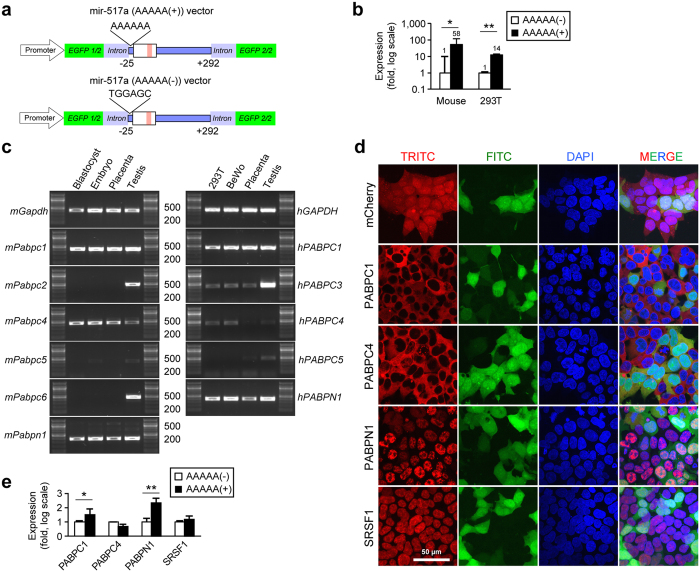
The binding of PABPs to miRNA intronic AAAAA. (**a**) A schematic depiction of forward-mir-517a (AAAAA(+)) or (AAAAA(−)) constructs. (**b**) A comparison between the expression level of forward-mir-517a (AAAAA(+)) and (AAAAA(−)) in mouse placenta (n = 5–15) and 293T cells (n = 3), determined by qPCR. (**c**) The expression of PABP family members in mouse or human tissues and cell lines, assessed using standard PCR. Mouse blastocysts were obtained at E3.5, embryos were obtained at E10.5, and placentas at E18.5. Human tissue included term placenta and adult testis. (**d**) 293T stable cell lines expressed mCherry (control) or mCherry-tagged PABPC1, PABPC4, PABPN1, or SRSF1. TRITC (red), FITC (green), and DAPI (blue) filter sets were used to detect mCherry, EGFP, and DAPI fluorophore, respectively. (**e**) RIP assays to determine the binding of relevant PABP proteins to the synthesized miR517-RNA ((AAAAA(+) or AAAAA(−)) construct, performed using RT-qPCR as described in Methods (n = 3 for each). *Denotes p < 0.05, **denotes p < 0.01.

We found that poly(A) (AAAAA) sites, which bind poly(A)-binding family proteins (PABP)^31^, are abundant among miRNA stem-flanking regions. For example, 85% (39/46) of C19MC miRNAs have AAAAA sequences within 500 nt of the 5′ flank (Fig. S13). To assess the potential role of AAAAA sequences within the pri-mir or pre-mir flanking sequences, we compared the expression levels of miR-517a-3p, which naturally harbours an AAAAA site within 25 bp upstream of the stem, with a construct in which we deleted the AAAAA site (Fig. 4a). As shown in Fig. 4b, the expression level of mir-517a (AAAAA(+)) was markedly higher than the expression level of mir-517a (AAAAA(−)) in the mouse placenta and in the 293T line *in vitro*. We surmised that the AAAAA motif binds members of the PABP family of proteins. To assess this possibility, we first determined which PABP family members are expressed in the mouse blastocyst. As shown in Fig. 4c, *Pabpn1, Pabpc1*, and *Pabpc4* mRNAs were expressed in the mouse blastocyst, and these genes were also expressed in the E10.5 embryo, E18.5 placenta, and adult mouse testis. A similar pattern was seen in human placental *PABPN1*, *PABPC1*, and *PABPC4* (Fig. 4c). To assess whether or not the relevant PABP members bind the AAAAA site, we stably expressed *Pabpn1, Pabpc1, Pabpc4*, as well as *Srsf1* (serving as a control RNA binding protein) in 293T cells. As expected^32^,^33^, SRSF1 and PABPN1 were appropriately localized to the nucleus, and PABPC1 and PABPC4 were localized to the cytoplasm (Fig. 4d). We transfected synthetic (AAAAA(+)) or (AAAAA(−)) mir-517a RNA into the PABP-expressing 293T cells and used RNA immunoprecipitation (RIP) to determine the binding of PABPs to the AAAAA at −13 nt from the 5′ end of mir-517a (MI0003161 in miRBase). As shown in Fig. 4e, we found that PABPC1 and PABPN1 bound the (AAAAA(+)) mir-517a RNA, supporting the notion that PABP binding to the Poly(A) site might reduce the likelihood of premature splicing prior to genomic integration.

### The overexpression of miRNA constructs in the mouse placenta

**Figure 5:**
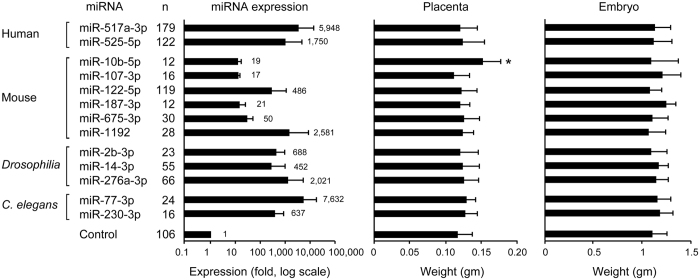
The efficiency of trophoblast-specific miRNA overexpression in the mouse placenta and its effect of on foeto-placental weight. Thirteen native and foreign miRNAs were overexpressed using our trophoblast lineage-specific miRNA expression strategy, and expression level was determined using RT-qPCR and shown with fold compared to eGFP-intron empty vector as control, using a log scale. The effect of each miRNA overexpression on placental weight or foetal weight is shown. *Denotes p < 0.05.

We used our transduction system to assess our ability to overexpress native or foreign miRNA constructs in the mouse placenta *in vivo*. We overexpressed 13 different human, mouse, *Drosophila*, and *C. Elegans* miRNA constructs, using a total of 1379 embryos and placentas. We analysed the effect of overexpression on foetal and placental weight when (a) the litter contained at least 4 mice, and (b) there was at least a 100-fold increase in mature miRNA expression level for exogenous miRNAs (mir-517a, -525, -2b, -14, -276a, -77, -230) or at least a 10-fold increase for endogenous miRNAs (mir-10b, -107, -122, -187, -675, -1192). The expression level of each overexpressed miRNA is shown in Fig. 5 (left panel). Notably, our analysis showed no foetal abnormalities, and standard H&E staining revealed no detectable histological differences among the placentas. Interestingly, while we found no significant impact of miRNA overexpression on embryo weight (Fig. 5, right panel), placentas overexpressing miR-10b-5p were larger (Fig. 5, centre panel). Using cDNA microarrays, followed by PCR validation, we failed to detect a distinctly dysregulated target for miR-10b-5p (not shown).

## Discussion

In this study, we sought to design lentiviral vectors for efficient expression of mature miRNA *in vivo*. We assessed several design parameters, some of which have been previously assessed *in vitro* (see below), based on expression of an eGFP-intron splicing plasmid^19^ applied to expression in the mouse placenta. Our data indicate that the length of the arms flanking the miRNA stem loop is an important determinant of expression efficiency, with longer arms supporting a higher level of miRNA expression. This observation is consistent with the notion that the proper activity of RNA-processing proteins depends on RNA length^34^,^35^. We also validated *in vivo* the strategy previously tested *in vitro* by Cooper *et al.*^20^ and showed that the reverse sense orientation strategy, designed to prevent pre-integration intron splicing, was highly effective in the mouse placenta. Not surprisingly, the effect of the flanking arms on expression efficiency was particularly prominent using our “forward sense lentivector” design, when intron length was less than 400 nt, and was minimized by the more effective “reverse sense lentivector” design.

Within the flanking pre-miRNA arms, we identified the PABP binding site, AAAAA, as an important determinant of miRNA expression efficiency. PABP proteins are known to bind the poly(A)-tail of mRNAs and bolster mRNA stability and translation^31^,^36^,^37^. Among members of the PABP family of proteins, we found that PABPC1, PABPC4, and PABPN1 are expressed in the mouse blastocyst and in both the mouse and human placenta. Using RIP, we showed that PABPC1 and PABPN1 directly bind a mir-517a (AAAAA(+)) fragment, but not a mir-517a (AAAAA(−)) fragment. Taken together, these data suggest that the PABP binding (AAAAA) site protects the miRNA-harbouring introns from premature splicing prior to genomic integration^38^,^39^,^40^. Interestingly, we noted that the expression level of human C19MC miRNAs that contain at least one AAAAA site in their flaking arms is higher than the expression of C19MC miRNAs lacking AAAAA (not shown). While distinctive mechanisms for miRNA processing and splicing may occur in different tissues, our data add to existing information on flanking arm sequences that are critical for miRNA processing^1^,^2^,^41^.

The general configuration of pri-miRNA from lower organisms, such as *drosophila* and *C. elegans*, is similar to that of higher organisms such as mice and humans, yet the size of mammalian miRNA introns (1000–2000 nt in the mouse and human) is generally longer than that of *drosophila* and *C. elegans and*, (500 and 300 nt, respectively,^42^,^43^. Interestingly, the expression of *drosophila* and *C. elegans* miRNA *in vitro* or *in vivo* in mammalian cells is fairly inefficient^1^. We observed this inefficient expression using our pre-mir-vector for expression of the *C. elegans*–specific miRNAs (mir-77, mir-230) in the mouse placenta. To overcome this hurdle, we engineered a “cassette” vector based on the structure of mir-517a, one of the most abundant miRNA in human placenta^9^. Replacing the mature miR-517 “cassette” with that of the *C. elegans*’s mature mir-77 or mir-230, we achieved a high expression level of these *C. elegans* miRNAs in the mouse placenta *in vivo*. These data suggest that the use of a cassette vector may enable the efficient expression of diverse miRNA types in mammalian cells irrespective of species-specific regulatory elements that are located outside the mature miRNA stem sequences.

Using a lentivector-based, placenta-specific miRNA expression system, we were able to efficiently evaluate diverse factors that may govern efficient miRNA processing *in vivo*. Notably, the trophectoderm, and, later, the placental trophoblasts, are particularly resilient to lentivirus-based transduction, when compared to a two-cell-stage embryo^24^. This may be explained by the position of the trophectoderm and, later, the placental trophoblast as the first line of foeto-placental defence against invading viral pathogens. Consistent with this notion, we have previously reported that primary human trophoblasts are markedly more resistant to viral infection than non-trophoblastic cells, and this mechanism involves C19MC miRNAs^44^. Our data suggest that the trophectoderm has a potent cytoplasmic or nuclear splicing system, which protects the inner cell mass from ssRNA viruses, particularly when producing short introns (<400 nt).

Using our system, we showed that we could successfully overexpress a total of 13 miRNAs (6 endogenous and 7 exogenous miRNA species) selectively in the trophoblast lineage of the mouse embryo. We chose these 13 miRNAs on the basis of species diversity, and their putative functions in processes such as cancer and metabolism. Interestingly, one of the miRNAs we tested, miR-675, has been previously shown to restrict murine placental size, with oversized placentas in mice deficient in miR-675^45^. We found that 50-fold overexpression of miR-675-3p had no effect on placental weight, suggesting that overexpressed miR-675 target(s) might regulate placental growth, but that underexpression of putative miR-675 target(s) had no phenotypic effect. Overall, the absence of a clear phenotype as a result of miRNA overexpression might reflect the notion that many miRNAs participate in homeostatic regulation of complex and redundant pathways. Additional molecular manipulations, coupled with altered physiologic conditions, might be needed to precisely define miRNA function. Nonetheless, we believe that our trophoblast lineage-specific miRNA overexpression system may be instrumental in defining the function of miRNA species during foeto-placental development, and assess trophoblast development and function.

## Methods

### Mouse experiments

Our experiments were approved by the Institutional Animal Care and Use Committee of the University of Pittsburgh (protocol number 13092512) and conducted in accordance with United States Public Health Service (PHS) Policy, as defined in the *Guide for the Care and Use of Laboratory Animals* prepared by the National Academy of Sciences. CD1 (ICR) mice, including vasectomized males, were purchased from Charles River Laboratories (Wilmington, MA). Mice were kept under constant conditions with standard rodent chow and water *ad libitum* on a 12:12 h light-dark cycle in room air. Timed matings were carried out by evening pairing of males and females, with the next morning after mating designated as embryonic day 0.5 (E0.5). Pregnant mice were euthanized by CO_2_ asphyxiation, and abdominal delivery was performed immediately. All placentas were observed for EGFP signal using a fluorescent dissecting microscope (SZX9, Olympus, Tokyo, Japan). Embryos and placentas (mean 4–14 per litter) were weighed and used for analysis. Genomic DNA was extracted from embryo tails by the alkaline lysis and boiling method^46^, and the sex was determined using standard PCR, as previously described^47^,^48^.

### Lentiviral DNA plasmids

For lentiviral plasmid DNA reconstruction, the following reagents and kits were used: PrimeSTAR GXL DNA polymerase (R050A, TaKaRa Bio, Tokyo, Japan), restriction enzymes (New England BioLabs, Ipswich, MA), T4 DNA ligase (M0202L, New England BioLabs), GenCatch PCR Purification Kit (2360250, Epoch Life Science, Sugar Land, TX), and QIAquick Gel Extraction Kit (28704, Qiagen, Hilden, Germany). All DNA oligonucleotides were purchased from IDT (Integrated DNA Technologies, Coralville, IA) and are listed in Supplementary Table S1. The design of each DNA plasmid is shown in Figs S2–12 and described below:

- *Promoter modification in the expression cassettes*: The Ubi-C promoter of the FUGW (Addgene #14883)^49^ plasmid was replaced by the more potent EF1a promoter (Addgene #11154) between the PacI and BamHI sites^50^,^51^,^52^. This lentiviral plasmid was termed Forward-EF1a-EGFP/FUGW. The reconstruction workflow is shown in Fig. S2, and the whole plasmid sequence is shown in Supplementary Materials Supplementary Sequence S1.
- *eGFP-intron splicing system*: We used the eGFP-intron splicing system to overexpress miRNA, as previously described^19^. EGFP in the original FUGW was replaced by an eGFP-intron cassette between BamHI and EcoRI. Our eGFP-intron cassette has two BsmBI sites for cloning with XhoI/NotI sites after digestion. This lentivector was named EF1a-eGFP-intron/FUGW, and the sequence is shown in Supplementary Sequence S2. A schematic of the eGPF-intron splicing system for miRNA expression is shown in Fig. 1b. The reconstruction workflow is shown in Figs S3 and S4, and the whole plasmid sequence is shown in Supplementary Sequence S1.
- *Primary (pri) mir-vector and precursor (pre) mir-vector*: Information regarding miRNA stem-loop structure and genome context were obtained from miRBase. PCR products were obtained, including the stem-loop with extension in both the 5′ and 3′ directions. After digestion with XhoI and NotI, the PCR products were ligated into the Forward-EF1a-eGFP-intron/FUGW plasmid. When the extension was approximately 200–300 nt, the lentivector was termed a “pri-mir-vector,” and when the extension was 25 nt, the lentivector was termed a “pre-mir-vector.” The pre-/pri-mir-vector is shown schematically in Fig. 1c. The reconstruction workflow is shown in Fig. S5.
- *Forward vector/Reverse vector*: Lentiviruses contain a single stranded RNA genome, and the expression cassette is positioned in a positive-sense orientation in most lentiviral plasmids, including FUGW. To prevent splicing prior to integration in the genome, we inserted the expression cassette in a negative-sense direction, as shown by Cooper *et al.*^20^. The expression cassette of EF1a promoter/eGFP-intron/WPRE was inserted in both orientations, positive-sense or negative-sense, between PacI and KpnI in the original FUGW. These lentivectors were termed the forward- and reverse-vectors, respectively. The schematic explanation of reverse-vector and forward-vector is shown in Fig. 1d. The reconstruction workflow is shown in Fig. S6, and the whole plasmid sequence of reverse-EF1a-eGFP-intron/FUGW is shown in Supplementary Sequence S3.
- *Frame vector*: Although miR-155 and miR-30 were previously used as frame vectors^17^,^18^, we preferred to use miR-517a because the primate-specific miR-517a is most abundant in the human placenta^9^. The schematic explanation of the frame vector is shown in Fig. 2a. The reconstruction workflow is shown in Figs S7–S9, and the whole plasmid sequence of reverse-EF1a-eGFP-intron-frame/FUGW is shown in Supplementary Sequence S4.
- *Truncated vectors*: Truncated mir-517a/mir-525 vectors were reconstructed in the same manner as the pre-/pri-vectors in the Forward EF1a-eGFP-intron/FUGW. A schematic depiction of the truncated vectors is shown in Fig. 3b,c. The reconstruction workflow is shown in Fig. S5.
- *mir-517a (AAAAA(−))/mir-517a (AAAAA(*+*)) vector*: The mir-517a (AAAAA(−)) and mir-517a (AAAAA(+)) vectors were constructed as described for the pre-/pri-mir-vectors in the Forward-EF1a-eGFP-intron/FUGW. A schematic design is depicted in Fig. 4a. The reconstruction workflow is shown in Fig. S10.
- *PABP family expression vector*: The PABP family expression vectors *Pabp1, Pabpc1,* and *Pabpc4*, as well as *Srsf1* as a negative control, were constructed by fusing the coding sequence with mCherry at the 3′ end, using a flexible amino acid linker (GGGGSGGGGS)^53^. Constructs were driven by the EF1a promoter. A schematic design of the vectors is shown in Fig. 4d. The reconstruction workflow is shown in Fig. S11.

### Lentivirus production and titration

Lentiviral expression plasmids were transfected, along with the envelope plasmid (pLTR-G, Addgene #17532) and the packaging plasmid (pCD/NL-BH*DDD, Addgene #17531), into 293T cells, using polyethylenimine, and the virus-containing culture media were harvested at 48 and 72 h. The culture media were centrifuged (500 g, 10 min), filtered through a 0.2 μm syringe filter (6780-2502, Puradisc, Whatman, Maidstone, United Kingdom), ultracentrifuged twice (50,000 g, 2 h each), and finally concentrated by 400-fold and resuspended in DPBS. The viral titer was determined using 293T cells.

### Trophoblast lineage-specific gene manipulation in the mouse placenta

Trophoblast lineage-specific gene manipulation procedures using lentiviruses have been previously described^24^. Before their mating with male mice, superovulation of females was performed by injecting 5 units of pregnant mare serum gonadotropin (G4877, Sigma, St. Louis, MO), followed by 5 units of human chorionic gonadotropin (C1063, Sigma) 48 h later. Blastocysts were harvested at E3.5 by flushing the uterine cavity with FHM medium (MR-024-D, Millippore, Bellerica, MA), and the zona pellucida was removed using acidic Tyrode’s solution (T1788, Sigma). After a few washes in KSOM medium (MR-121-D, Millipore), zona-free blastocysts were incubated for 5 h with the lentivirus, at the concentration of 1 × 10^7^ TU/mL in 10 μL of CO_2_-equillibrated KSOM medium. After anaesthesia using Avertin, lentivirus-exposed zona-free blastocysts were transferred into E2.5 pseudopregnant female mice. Abdominal delivery was performed 15 days after embryo transfer (E18.5), and the tissues were analysed.

### Total RNA extraction

Total RNA was isolated from mouse blastocysts, E10.5 embryos, placentas (E14.5/E18.5), adult testes, and cell lines (293T or BeWo, both from ATCC, Manassas, VA), using TRI Reagent (Molecular Research Center, Cincinnati, OH) according to the manufacturer’s instruction, and the quality and quantity of total RNA were assessed by 260/280 and 260/230 absorbance ratio, using a NanoDrop 1000 spectrophotometer (Thermo Scientific, Wilmington, DE). Total RNA samples from human organs were purchased from Ambion (Austin, CA).

### RT-qPCR for miRNAs

cDNA synthesis and qPCR were performed using miScript PCR system (Qiagen) and ViiA 7 Real Time PCR system (Applied Biosytems, Foster City, CA) according to the manufacturer’s instructions. RNU6B was used for normalization, and the fold change relative to control samples was determined by the 2^−ΔΔCt^ method^54^. All of the miScript primer pairs are listed in Supplementary Table S2.

### Regular RT-PCR/qPCR for mRNA

cDNAs were synthesized using the High Capacity cDNA Reverse Transcription Kit (Applied Biosytems) according to the manufacturer’s instructions. Standard RT-PCR was carried out using KOD Xtreme DNA polymerase (EMD, Gibbstown, NJ) in a Veriti thermal cycler (Applied Biosytems), and electrophoresed using ethidium bromide, prestained 1.2% TAE agarose gel at 100V for 20 min. For *q*PCR, the ViiA 7 Real-Time PCR System (Applied Biosystems) was used with SYBR Green PCR master mix (Applied Biosystems) and GAPDH for normalization. The specificity of amplification of the PCR product was confirmed using a single-peaked dissociation curve. All primer pairs used for regular RT-PCR are listed in Supplementary Table S3.

### Histological and immunohistochemical analysis

For standard histology, placentas were harvested, fixed overnight in 4% paraformaldehyde in DPBS, and embedded in paraffin using standard procedures. Paraffin sections (5 μm) were cut using a microtome (Leica RM2255, Wetzlar, Germany) and stained with SelecTech Hematoxylin and Eosin Staining System (Surgipath, Richmond, IL). A Nikon 90i microscope (Nikon, Tokyo, Japan) was used to examine for H&E stained sections, an Axiovert 40 CFL inverted microscope (Carl Zeiss, Oberkochen, Germany) was used for blastocyst imaging, and a Nikon A1 confocal microscope was used for cultured cells. For detection of GFP we used immunohistochemical staining of paraffin-embedded sections, processed from 4% paraformaldehyde-fixed placentas using standard procedures. GFP was detected using rabbit polyclonal anti-GFP antibody (1:100, #ab290 Abcam, Cambridge MA, USA), and the signal was detected using Vectastain Elite ABC HRP Kit (PK-6101, Vector Laboratories, Burlingame, CA, USA).

### RNA immunoprecipitation (RIP)

PABP family expression lentivirus was used for 293T transduction, and stable lines were established using limiting dilution cultures. PCR products were obtained from mir-517a (AAAAA(+))/(AAAAA(−)) vectors as template, using the following primer pair: forward (T7 RNA polymerase site)-(EGFP forward); GAAATTAATACGACTCACTATA-ACGTAAACGGCCACAAGTTC, reverse (EGFP reverse); GTCCTCCTTGAAGTCGATGC. Synthetic RNA (AAAAA(+) or AAAAA(−)) was obtained by *in vitro* reaction with PCR products and T7 RNA polymerase (Roche, Basal, Switzerland) according to the instruction manual. After DNaseI (Roche) digestion, the quality and quantity of synthetic RNAs were assessed using a Nanodrop 1000 spectrophotometer. Synthetic RNAs were transfected into the stable 293T cell lines using Lipofectamine 2000 (Invitrogen, Carlsbad, CA). RIP was performed 24 h after transfection, using RIP-Assay Kit (RN1001, MBL, Tokyo, Japan), and the extracted RNA samples were analysed by RT-qPCR. The following primer pairs were used: synthetic RNA, forward; ACGACGGCAACTACAAGACC, reverse; TCTCGACAAGCCCAGTTTCT, GAPDH, forward; GAAGGTCGGAGTCAACGGATTT, reverse; GAATTTGCCATGGGTGGAAT.

### Statistics

Statistical analysis was performed using analysis of variance, with the Boneferroni *post hoc* test for multiple comparisons of placental and embryo weight, and Student’s t-test for RT-qPCR using Dr. SPSS II (version11) for Windows (SPSS, Chicago, IL). Significance was determined at p < 0.05.

## Additional Information

**How to cite this article**: Mishima, T. *et al.* Determinants of effective lentivirus-driven microRNA expression *in vivo. Sci. Rep.* **6**, 33345; doi: 10.1038/srep33345 (2016).

## Supplementary Material

Supplementary Information
