# Supplementary material for: Determinants of effective lentivirus-driven microRNA expression in vivo
Source: Sci Rep. 2016 Sep 15;6:33345. doi: 10.1038/srep33345 (PMC5024309; doi:10.1038/srep33345)
Supplement: Supplementary Information [file srep33345-s1.pdf]

SUPPLEMENTARY INFORMATION

Determinants of effective lentivirus-driven microRNA expression *in vivo*

Takuya Mishima, Elena Sadovsky, Margaret E. Gegick, Yoel Sadovsky

Supplementary information for this manuscript includes the following:

- 13 Figures
- 4 Sequence documents
- 3 Tables

(A) Statistics

Pregnant mice after embryo transfer	**186**
Abdominal delivery on E18.5	**181**
Transferred blastocysts	**2998**
Live pups with EGFP positive placenta	**1379**
Pups per Blastocyst rate (1379/2998)	**46.0%**
Pups per pregnant mouse (1379/181)	**7.62**
Blastocysts per pregnant mouse (2998/181)	**16.6**

	Male	Female	M/F ratio
Embryo Count	**196**	**150**	**1.30**

*Significant difference according to Chi-Square Test P=.0134

(B) Regular RT-PCR (EGFP)

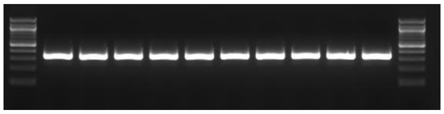

(C) PCR of genome DNA (EGFP)

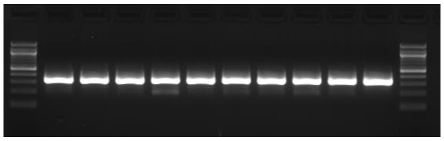

Supplementary Figure S1. (A) Summary of experimental procedures used in trophoblast lineage specific gene manipulation. (B) Regular RT-PCR of EGFP mRNA in mouse placenta after trophoblast lineage specific gene manipulation. PCR cycle number was 30, and PCR product size is 338bp. (c) PCR of EGFP for genome DNA in mouse placenta after trophoblast lineage specific gene manipulation. PCR cycle number was 30, and PCR product size is 338bp. Forward primer; ACGTAAACGGCCACAAGTTC, Reverse primer; GTCCTCCTTGAAGTCGATGC.

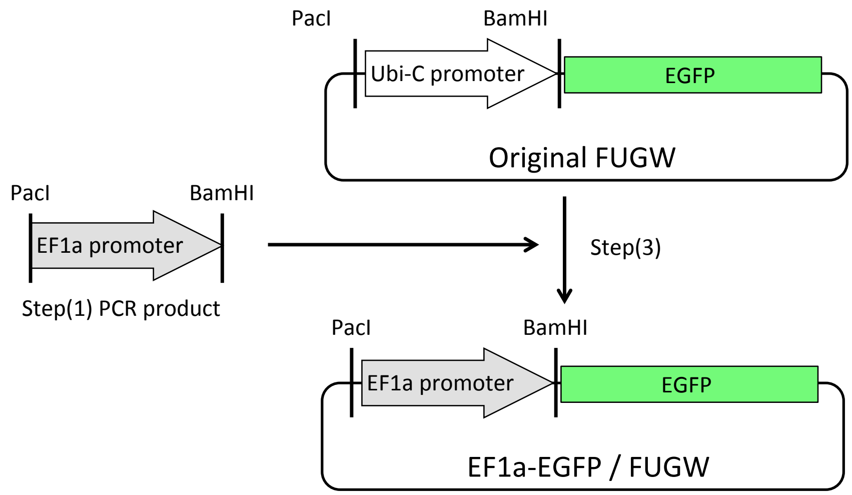

Supplementary Figure S2. Schematic workflow of lentiviral DNA plasmid (i) Forward-EF1a-EGFP / FUGW.

Step (1) PCR (EF1a promoter), PCR clean-up, Enzyme Digestion (PacI/BamHI), PCR cleanup
Template: Human Genome DNA
(PacI)-(EF1a forward): CCttaattaaCGTGAGGCTCCGGTGC
(BamHI)-(EF1a reverse): CGCggatccCTCACGACACCTGAAATGGAAG

Step (2) Enzyme Digestion of original FUGW (PacI/BamHI), Gel extraction kit

Step (3) Ligation step (1) and step (2), and transformation

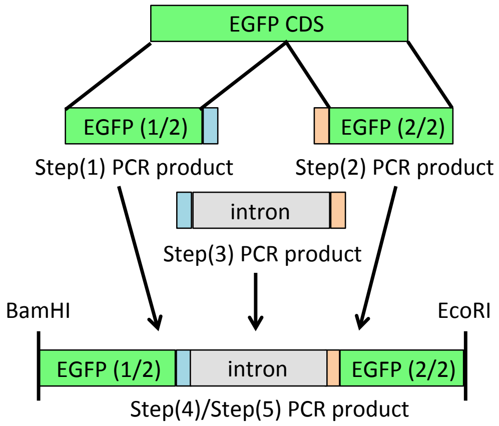

Supplementary Figure S3. Schematic workflow of lentiviral DNA plasmid (ii) Forward-EF1a-eGFP-intron / FUGW (Steps (1)-(5)).

Step (1) PCR (EGFP(1/2)), PCR clean-up
Template: original FUGW pDNA
(BamHI)-(Kozak)-(EGFP forward from start codon):
CGCggatccGCCACCATGGTGAGCAAGGGCGA
(Intron-eGFP antisense): GTGTCGCCCTCGAACTTCACCTGTGGAGAGAAAGGCA

Step (2) PCR (EGFP(2/2)), PCR clean-up
Template: original FUGW pDNA
(intron-eGFP sense): ACTACAAGACCCGCGCCGAGGTAAGTATCAAGGTTAC
(EcoRI)-(EGFP reverse from stop codon): CCGgaattcTTACTTGTACAGCTCGTCCATGC

Step (3) PCR (synthesized intron) (5-cycles), PCR clean-up
Template: (-)
Chemically-synthesized intron forward (BsmBI x2):
GTAAGTATCAAGGTTACAAGACAGGTTTAAGGAGACCAATAGAAACTGGGCTTGTCGAG
ACAGAGAAGACTCTTGCGTTTTCGATGAGAC
Chemically-synthesized intron reverse (BsmBI x2):
CTGTGGAGAGAAAGGCAAAGTGGATGTCAGTAAGACCAATAGGTGCCTATCATGGCCTG
AGACGCATCTGTACGTCTCATCGAAAACGCA

Step (4) PCR (eGFP-intron) (5-cycles), PCR clean-up
Mix PCR products Step (1)-(3), and PCR

Step (5) PCR (eGFP-intron) (30-cycles), PCR clean-up
Template: Step (4) PCR product
(BamHI)-(Kozak)-(EGFP forward from start codon):
CGCggatccGCCACCATGGTGAGCAAGGGCGA
(EcoRI)-(EGFP reverse from stop codon): CCGgaattcTTACTTGTACAGCTCGTCCATGC

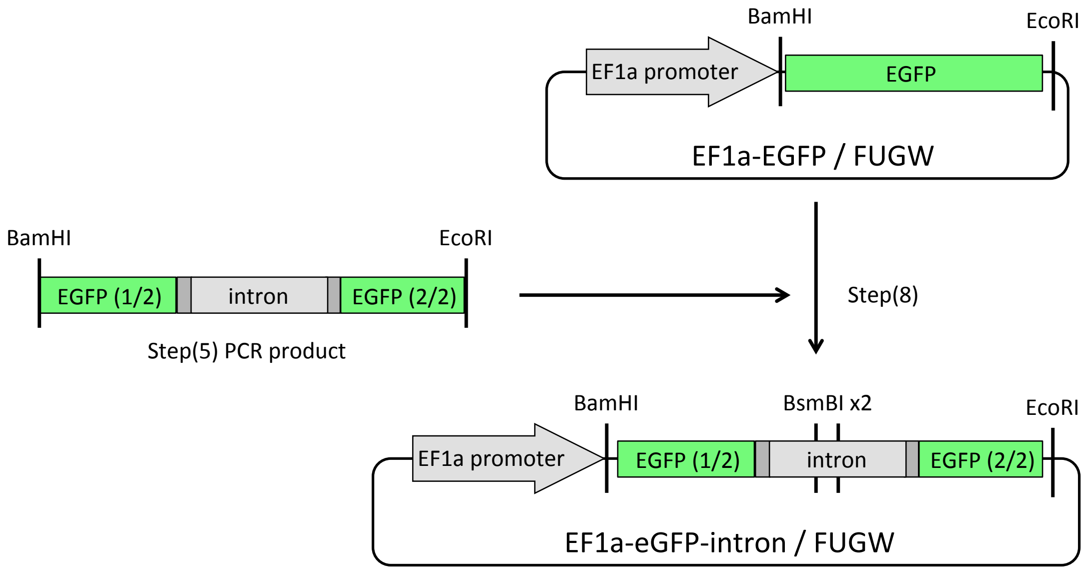

Supplementary Figure S4. Schematic workflow of lentiviral DNA plasmid (ii) Forward-EF1a-eGFP-intron / FUGW (Steps (6)-(8)).

Step (6) Enzyme digestion (BamHI/EcoRI) of PCR product in Step (5), PCR clean-up

Step (7) Enzyme digestion (BamHI/EcoRI) of EF1a-EGFP / FUGW, Gel extraction kit

Step (8) Ligation step (6) and step (7), and transformation

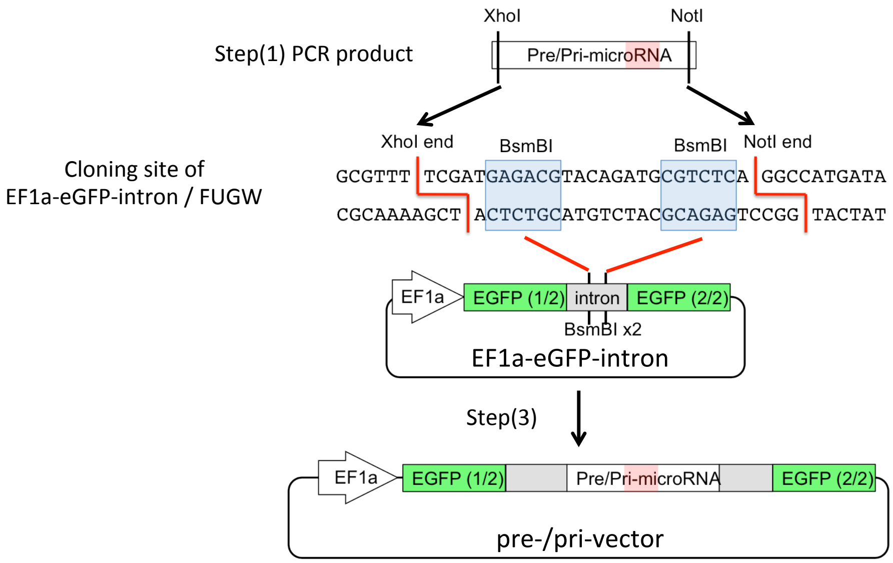

Supplementary Figure S5. Schematic workflow of lentiviral DNA plasmid; (iii) pre- / pri-mir-vector, (vi) truncated pri-mir-vector.

Step (1) PCR for pre- / pri-vector, PCR clean-up, Enzyme digestioin (XhoI/NotI), PCR clean-up
Template: each species or no template
Forward primer : See Supplementary Table S1 (iii)-1 pre-vector or (iii)-2 pri-vector primers
Reverse primer : See Supplementary Table S1 (iii)-1 pre-vector or (iii)-2 pri-vector primers

Step (2) Enzyme digestion (BsmBI) of EF1a-eGFP-intron / FUGW, PCR clean-up

Step (3) Ligation Step (1) and Step (2), and transformation

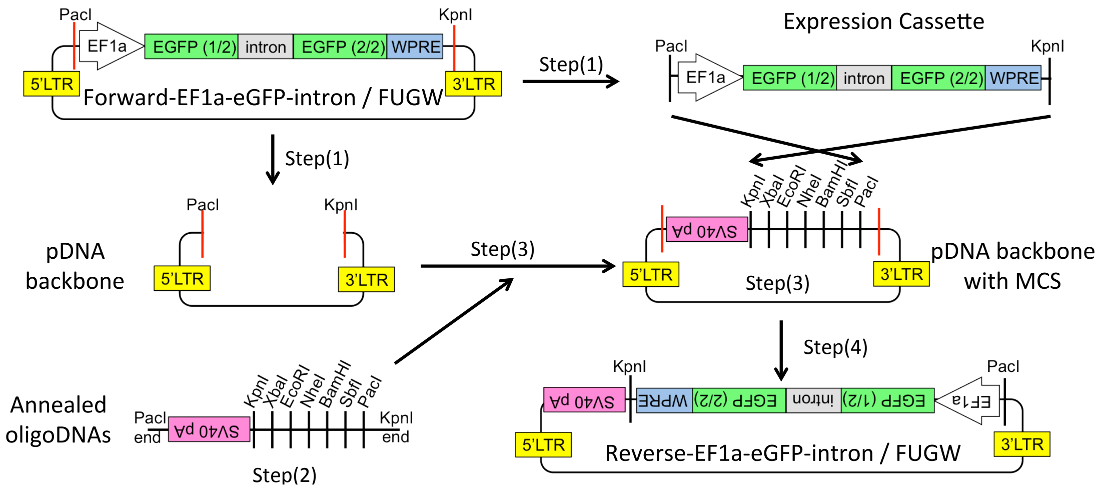

Supplementary Figure S6. Schematic workflow of lentiviral DNA plasmid; (iv) Reverse vector.

Step (1) Enzyme digestion (PacI/KpnI) of Forward-EF1a-eGFP-intron / FUGW, Gel extraction (pDNA backbone / Expression Cassette)

Step (2) Anneal the oligoDNAs for MCS (multiple cloning sites)
(SV40 pA Rev)-(KpnI)-(XbaI)-(EcoRI)-(NheI)-(BamHI)-(SbfI)-(PacI)-(KpnI End)
acaaaccacaactagaatgcagtgaaaaaaatgctttattggtaccTCTAGAgaattcGCTAGCggatccCCTGCAGGttaattaaGTAC
(PacI)-(SbfI)-(BamHI)-(NheI)-(EcoRI)-(XbaI)-(KpnI)-(SV40 pA)-(PacI end)
ttaattaaCCTGCAGGggatccGCTAGCgaattcTCTAGAggtaccaataaagcatttttttcactgcattctagttgtggtttgtAT

Step (3) Ligation Step (1)-(pDNA backbone) and Step (2), and transformation

Step (4) Insert Step (1)-(Expression cassette) between KpnI and PacI of Step (3) pDNA backbone with MCS

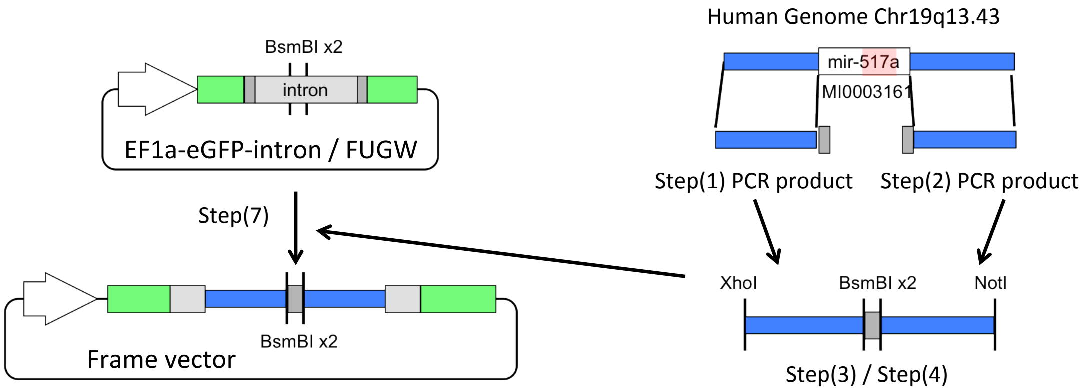

Supplementary Figure S7. Schematic workflow of lentiviral DNA plasmid; (v) Frame vector (Steps (1)-(7)).

Step (1) PCR for pri-mir-517a anterior, PCR clean-up
Template : Human genome DNA
(XhoI)-(pri-mir-517a forward)
aaccgctcgaggagtgcatggggtttgagtt
(BsmBI)-(BsmBI)-(pri-mir-517a anterior reverse)
gagacgcatctgtacgtctcctgcctgagatcttcttttttttg

Step (2) PCR for pri-mir-517a posterior, PCR clean-up
Template : Human genome DNA
(BsmBI)-(BsmBI)-(pri-mir-517a posterior forward)
gagacgTACAGATGcgtctcTACTGTTTGAGAcaagcaacgttg
(NotI)-(pri-mir-517a reverse)
gtagcggccgcgaggtgggagaatcactgga

Step (3) PCR to combine anterior and posterior
Mix PCR products from PCR(1) and PCR(2), and do PCR for 5 cycles

Step (4) PCR to amplify (pri-mir-517a anterior)-(BsmBI)-(BsmBI)-(pri-mir-517a posterior)
Template : Step (3) PCR product
(XhoI)-(pri-mir-517a forward):aaccgctcgaggagtgcatggggtttgagtt
(NotI)-(pri-mir-517a reverse):gtagcggccgcgaggtgggagaatcactgga

Step (5) Enzyme Digestion (XhoI/NotI) of Step (4) PCR product, PCR clean-up

Step (6) Enzyme Digestion of EF1a-eGFP-intron / FUGW, PCR clean-up

Step (7) Ligation Step (5) and Step (6), and transformation

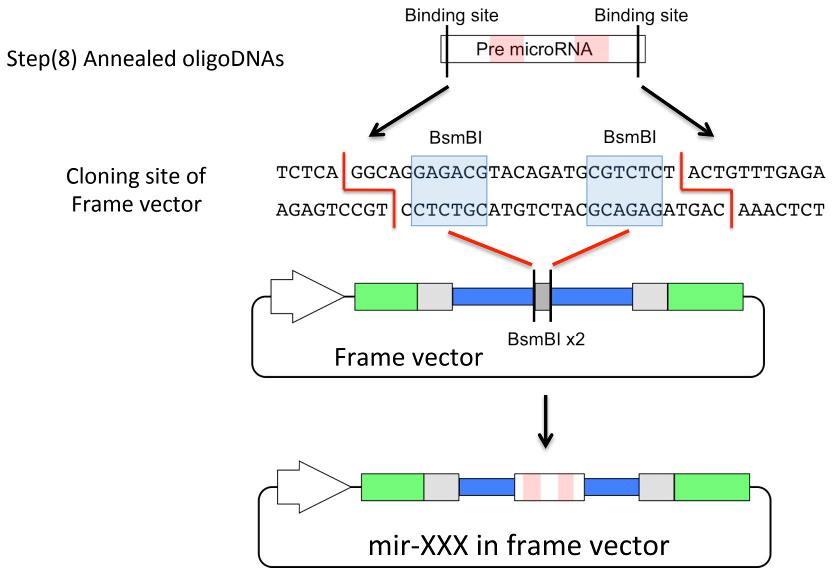

Supplementary Figure S8. Schematic workflow of lentiviral DNA plasmid; (v) Frame vector (Steps (8)-(10))

Step (8) Annealing the oligoDNAs for each miRNA (See Supplementary Table S1, (v) frame vector)

Step (9) Enzyme digestion (BsmBI) of Step (7) pDNA

Step (10) Ligation Step (8) and Step (9), and transformation

cel-mir-230 in frame

cel-mir-230

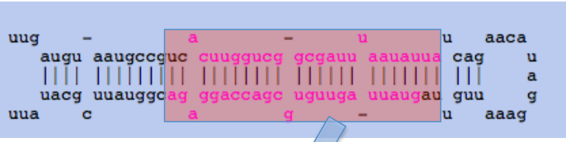

>cel-mir-230 MI0000305

TTGATGTAATGCCGTCACTTGGTCGGCGATTTAATATTATCAGAACATAGGAAATTGTTAGTATTAGTTGTGCGACCAGGAGACGGTATTCGCATATT

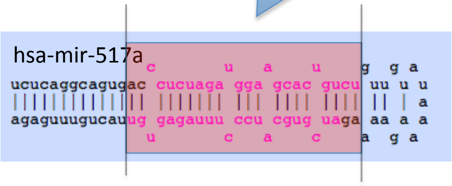

hsa-mir-517a MI0003161

UCUCAGGCAGUGACCCUCUAGAUGGAAGCACUGUCUGUUGUAUAAAAGAAAAGAUCGUGCAUCCCUUUAGAGUGUUACUGUUUGAGA

```
(5′ joint part)-(mir-230 5′arm)-(mir-517a loop)-(mir-230 3′arm)-(3′ joint part)
5′-ggcagtgTCACTTGGTCGGCGATTTAATATTAgttgtataaaagaaaTAGTATTAGTTGTGCGACCAGGAGAt-3′
3′-    cacAGTGAACCAGCCGCTAAATTATAATcaacatattttctttATCATAATCAACACGCTGGTCCTCTatgac-5′

TOP :      5′-ggcagtgTCACTTGGTCGGCGATTTAATATTAgttgtataaaagaaaTAGTATTAGTTGTGCGACCAGGAGAt-3′
Bottom :   5′-cagtaTCTCCTGGTCGCACAACTAATACTAtttcttttatacaacTAATATTAAATCGCCGACCAAGTGAcac-3′
```

Supplementary Figure S9. Schematic workflow of lentiviral DNA plasmid; (v) Frame vector (Example of annealed oligo DNA)

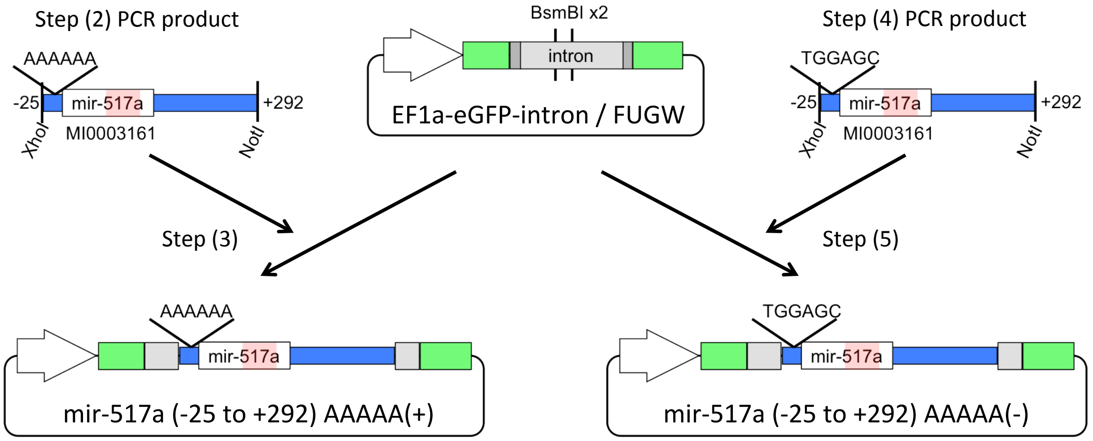

Supplementary Figure S10. Schematic workflow of lentiviral DNA plasmid; (vii) mir-517a (AAAAA(-)).

Step (1) Enzyme digestion (BsmBI) of EF1a-eGFP-intron / FUGW

Step (2) PCR (mir-517a AAAAA(+)), and enzyme digestion (XhoI/NotI)
Template : Human genome DNA
(XhoI)-(mir-517a forward) From -25 :
aaccgCTCGAGagactccgtgtcaaaaaaaagaaga
(NotI)-(pri-mir-517a reverse) From +292 :
gtaGCGGCCGCgaggtgggagaatcactgga

Step (3) Ligation Step (1) and Step (2), and transformation

Step (4) PCR (mir-517a AAAAA(-)), and enzyme digestion (XhoI/NotI)
Template : Human genome DNA
(XhoI)-(-25 to -14)-(In:TGGAGC, Out:AAAAAA)-(mir-517a forward) From -7 :
AACCGctcgagAGACTCCGTGTCtggagcAAGAAGATCTCAGGCAGTGACC
(NotI)-(pri-mir-517a reverse) From +292 :
gtaGCGGCCGCgaggtgggagaatcactgga

Step (5) Ligation Step (1) and Step (4), and transformation

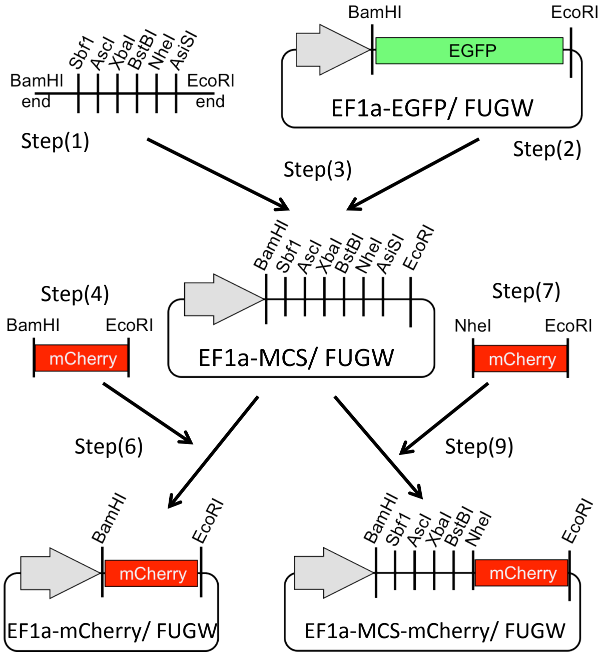

Supplementary Figure S11. Schematic workflow of lentiviral DNA plasmid; (viii) Pabp family expression vector (Steps (1)-(9))

Step (1) Annealing of oligoDNAs
(BamHI end)-(SbfI)-(AscI)-(XbaI)-(BstBI)-(NheI)-(AsiSI)-(EcoRI end)
GATCCCCTGCAGGGGCGCGCCTCTAGATTCGAAGCTAGCGGCGCGCCG
(EcoRI end)-(AsiSI)-(NheI)-(BstBI)-(XbaI)-(AscI)-(SbfI)-(BamHI end)
AATTCGGCGCGCCGCTAGCTTCGAATCTAGAGGCGCGCCCCTGCAGGG

Step (2) Enzyme Digestion (BamHI/EcoRI) of EF1a-EGFP/FUGW

Step (3) Ligation Step (1) and Step (2), and transformation

Step (4) PCR, and enzyme digestion(BamHI/EcoRI)
Template : CMV-Brainbow-1.1M (Addgene 18722)
(BamHI)-(Kozak)-(EGFP/mCherry/EBFP2 start)
CGCggatccGCCACCATGGTGAGCAAGGGCGA
(EcoRI)-(EGFP/mCherry/EBFP2 reverse from stop codon)
CCGgaattcTTACTTGTACAGCTCGTCCATGC

Step (5) Enzyme Digestion (BamHI/EcoRI) of EF1a-MCS / FUGW

Step (6) Ligation Step (4) and Step (5), and transformation

Step (7) PCR, and enzyme digestion (NheI/EcoRI)
Template : CMV-Brainbow-1.1M (Addgene 18722)
(NheI)-(EGFP/mCherry/EBFP2 forward from ATG)
AACATGgctagcATGGTGAGCAAGGGCGA
(EcoRI)-(EGFP/mCherry/EBFP2 reverse from stop codon)
CCGgaattcTTACTTGTACAGCTCGTCCATGC

Step (8) Enzyme Digestion (NheI/EcoRI) of EF1a-MCS / FUGW

Step (9) Ligation Step (4) and Step (5), and transformation

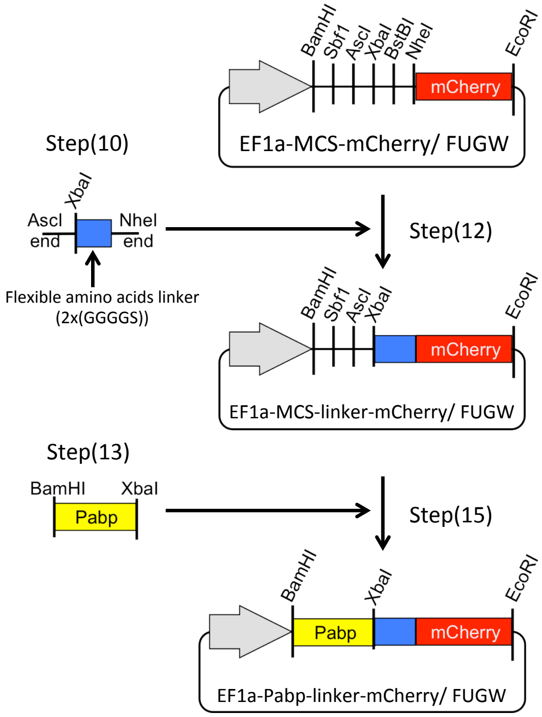

Supplementary Figure S12. Schematic workflow of lentiviral DNA plasmid; (viii) Pabp family expression vector (Steps (10)-(15)).

Step (10) Annealing of oligoDNAs
(AscI End)-(XbaI)-(2x GGGGS)-(NheI End)
CGCGCCtctagaGGCGGAGGTGGCAGCGGCGGAGGTGGCAGCg
(NheI End)-(2x GGGGS)-(XbaI)-(AscI End)
ctagcGCTGCCACCTCCGCCGCTGCCACCTCCGCCtctagaGG

Step (11) Enzyme Digestion (NheI/AscI) of EF1a-MCS-mCherry/FUGW

Step (12) Ligation Step (10) and Step (11), and transformation

Step (13) PCR, and enzyme digestion(BamHI/XbaI)
Template : mouse blastocyst cDNA
Forward primer : Supplementary Table1 (vii) Pabp family expression vector
Reverse primer : Supplementary Table1 (vii) Pabp family expression vector

Step (14) Enzyme Digestion (BamHI/XbaI) of EF1a-MCS-Linker-mCherry/FUGW

Step (15) Ligation Step (13) and Step (14), and transformation

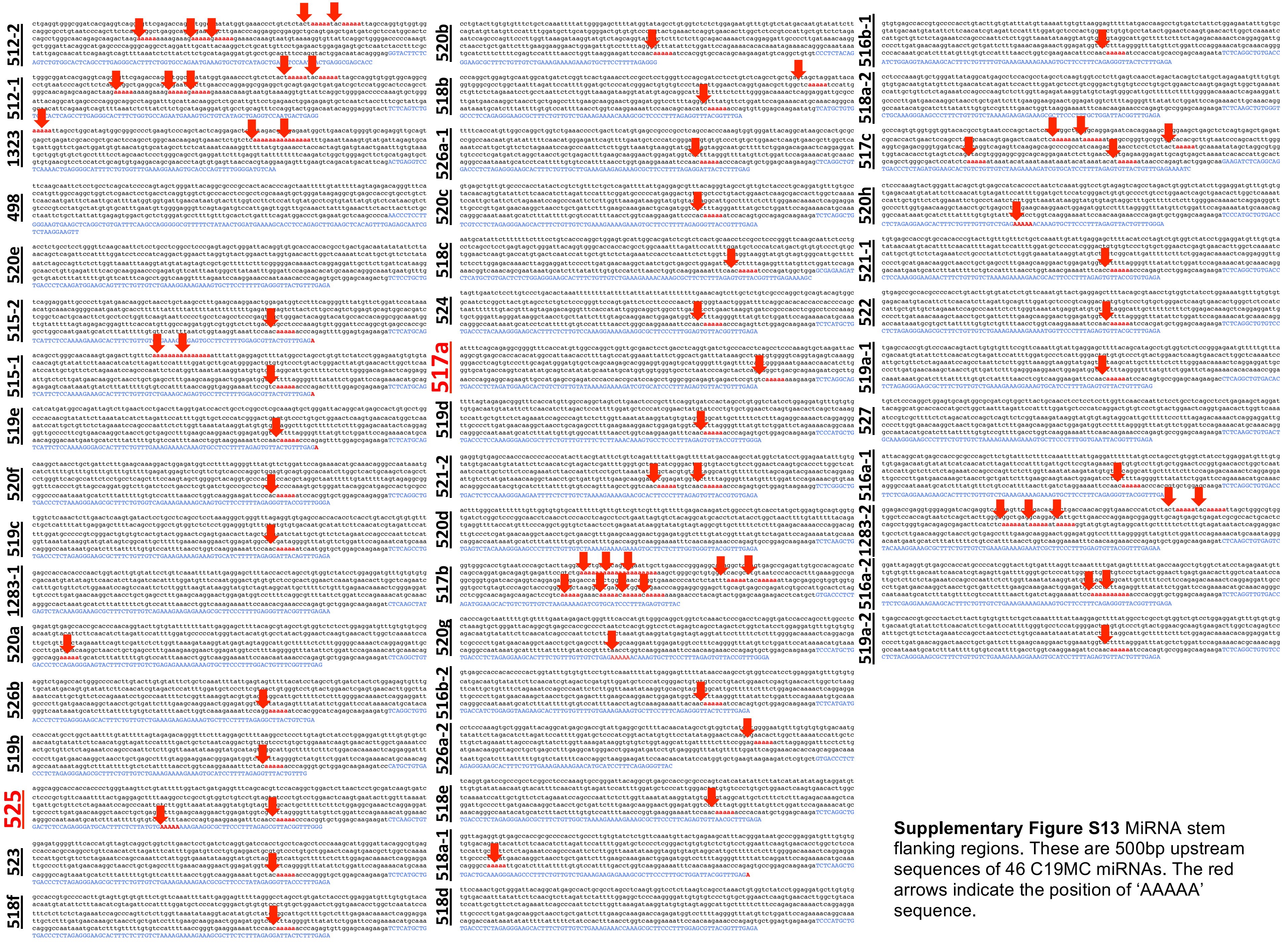

Supplementary Figure S13 MiRNA stem flanking regions. These are 500bp upstream sequences of 46 C19MC miRNAs. The red arrows indicate the position of 'AAAAA' sequence.

Lentiviral plasmid DNA reconstruction: Whole plasmid sequence 1

(i) Promoter for the expression cassette

Vector name : Forward-EF1a-EGFP / FUGW

Feature Name
CMV_immearly_promoter / HIV-1_5_LTR / truncHIV-1_3_LTR / HIV-1_psi_pack / RRE / cPPT / EF1a_promoter / EGFP / WPRE / U3PPT / HIV-1_5_LTR

GTCGACGGATCGGGAGATCTCCCGATCCCCTATGGTGCACTCTCAGTACAATCTGCTCTGATGCCGCATAGTTAAGCCAGTATCTGCTC
CCTGCTTGTGTGTTGGAGGTCGCTGAGTAGTGCGCGAGCAAAATTTAAGCTACAACAAGGCAAGGCTTGACCGACAATTGCATGAAGAA
TCTGCTTAGGGTTAGGCGTTTTGCGCTGCTTCGCGATGTACGGGCCAGATATACGCGTTGACATTGATTATTGACTAGTTATTAATAGT
AATCAATTACGGGGTCATTAGTTCATAGCCCATATATGGAGTTCCGCGTTACATAACTTACGGTAAATGGCCCGCCTGGCTGACCGCCC
AACGACCCCCGCCCATTGACGTCAATAATGACGTATGTTCCCATAGTAACGCCAATAGGGACTTTCCATTGACGTCAATGGGTGGAGTA
TTTACGGTAAACTGCCCACTTGGCAGTACATCAAGTGTATCATATGCCAAGTACGCCCCCTATTGACGTCAATGACGGTAAATGGCCCG
CCTGGCATTATGCCCAGTACATGACCTTATGGGACTTTCCTACTTGGCAGTACATCTACGTATTAGTCATCGCTATTACCATGGTGATG
CGGTTTTGGCAGTACATCAATGGGCGTGGATAGCGGTTTGACTCACGGGGATTTCCAAGTCTCCACCCCATTGACGTCAATGGGAGTTT
GTTTTGGCACCAAAATCAACGGGACTTTCCAAAATGTCGTAACAACTCCGCCCCATTGACGCAAATGGGCGGTAGGCGTGTACGGTGGG
AGGTC**TATATAA**GCAGCGCGTTTTGCCTGTACTGGGTCTCTCTGGTTAGACCAGATCTGAGCCTGGGAGCTCTCTGGCTAACTAGGGAA
CCCACTGCTTAAGCCTCAATAAAGCTTGCCTTGAGTGCTTCAAGTAGTGTGTGCCCGTCTGTTGTGTGACTCTGGTAACTAGAGATCCC
TCAGACCCTTTTAGTCAGTGTGGAAAATCTCTAGCAGTGGCGCCCGAACAGGGACTTGAAAGCGAAAGGGAAACCAGAGGAGCTCTCTC
GACGCAGGACTCGGCTTGCTGAAGCGCGCACGGCAAGAGGCGAGGGGCGGCGACTGGTGAGTACGCCAAAAATTTTGACTAGCGGAGGC
TAGAAGGAGAGAGATGGGTGCGAGAGCGTCAGTATTAAGCGGGGGAGAATTAGATCGCGATGGGAAAAAATTCGGTTAAGGCCAGGGGG
AAAGAAAAAATATAAATTAAAACATATAGTATGGGCAAGCAGGGAGCTAGAACGATTCGCAGTTAATCCTGGCCTGTTAGAAACATCAG
AAGGCTGTAGACAAATACTGGGACAGCTACAACCATCCCTTCAGACAGGATCAGAAGAACTTAGATCATTATATAATACAGTAGCAACC
CTCTATTGTGTGCATCAAAGGATAGAGATAAAAGACACCAAGGAAGCTTTAGACAAGATAGAGGAAGAGCAAAACAAAAGTAAGACCAC
CGCACAGCAAGCGGCCGCTGATCTTCAGACCTGGAGGAGGAGATATGAGGGACAATTGGAGAAGTGAATTATATAAATATAAAGTAGTA
AAAATTGAACCATTAGGAGTAGCACCCACCAAGGCAAAGAGAAGAGTGGTGCAGAGAGAAAAAAGAGCAGTGGGAATAGGAGCTTTGTT
CCTTGGGTTCTTGGGAGCAGCAGGAAGCACTATGGGCGCAGCGTCAATGACGCTGACGGTACAGGCCAGACAATTATTGTCTGGTATAG
TGCAGCAGCAGAACAATTTGCTGAGGGCTATTGAGGCGCAACAGCATCTGTTGCAACTCACAGTCTGGGGCATCAAGCAGCTCCAGGCA
AGAATCCTGGCTGTGGAAAGATACCTAAAGGATCAACAGCTCCTGGGGATTTGGGGTTGCTCTGGAAAACTCATTTGCACCACTGCTGT
GCCTTGGAATGCTAGTTGGAGTAATAAATCTCTGGAACAGATTTGGAATCACACGACCTGGATGGAGTGGGACAGAGAAATTAACAATT
ACACAAGCTTAATACACTCCTTAATTGAAGAATCGCAAAACCAGCAAGAAAAGAATGAACAAGAATTATTGGAATTAGATAAATGGGCA
AGTTTGTGGAATTGGTTTAACATAACAAATTGGCTGTGGTATATAAAATTATTCATAATGATAGTAGGAGGCTTGGTAGGTTTAAGAAT
AGTTTTTGCTGTACTTTCTATAGTGAATAGAGTTAGGCAGGGATATTCACCATTATCGTTTCAGACCCACCTCCCAACCCCGAGGGGAC
CCGACAGGCCCGAAGGAATAGAAGAAGAAGGTGGAGAGAGAGACAGAGACAGATCCATTCGATTAGTGAACGGATCGGCACTGCGTGCG
CCAATTCTGCAGACAAATGGCAGTATTCATCCACAATTTTAAAAGAAAAGGGGGGATTGGGGGGTACAGTGCAGGGGAAAGAATAGTAG
ACATAATAGCAACAGACATACAAACTAAAGAATTACAAAAACAAATTACAAAAATTCAAAATTTTCGGGTTTATTACAGGGACAGCAGA
GATCCAGTTTGGttaattaacgtgaggctccggtgcccgtcagtgggcagagcgcacatcgcccacagtccccgagaagttggggggag
gggtcggcaattgaaccggtgcctagagaaggtggcgcggggtaaactgggaaagtgatgtcgtgtactggctccgcctttttcccgag
ggtgggggagaaccgtatataagtgcagtagtcgccgtgaacgttctttttcgcaacgggtttgccgccagaacacaggtaagtgccgt
gtgtggttcccgcgggcctggcctctttacgggttatggcccttgcgtgccttgaattacttccacgcccctggctgcagtacgtgatt
cttgatcccgagcttcgggttggaagtgggtgggagagttcgaggccttgcgcttaaggagccccttcgcctcgtgcttgagttgaggc
ctggcctgggcgctggggccgccgcgtgcgaatctggtggcaccttcgcgcctgtctcgctgctttcgataagtctctagccatttaaa
atttttgatgacctgctgcgacgctttttttctggcaagatagtcttgtaaatgcgggccaagatctgcacactggtatttcggttttt
ggggccgcgggcggcgacggggcccgtgcgtcccagcgcacatgttcggcgaggcggggcctgcgagcgcggccaccgagaatcggacg
ggggtagtctcaagctggccggcctgctctggtgcctggcctcgcgccgccgtgtatcgccccgccctgggcggcaaggctggcccggt
cggcaccagttgcgtgagcggaaagatggccgcttcccggccctgctgcagggagctcaaaatggaggacgcggcgctcgggagagcgg
gcgggtgagtcacccacacaaaggaaaagggcctttccgtcctcagccgtcgcttcatgtgactccacggagtaccgggcgccgtccag
gcacctcgattagttctcgagcttttggagtacgtcgtctttaggttggggggaggggttttatgcgatggagtttccccacactgagt
gggtggagactgaagttaggccagcttggcacttgatgtaattctccttggaatttgccctttttgagtttggatcttggttcattc**tc**
aagcctcagacagtggttcaaagtttttttcttccatttcaggtgtcgtgaggatccGCCACCATGGTGAGCAAGGGCGAGGAGCTGT
TCACCGGGGTGGTGCCCATCCTGGTCGAGCTGGACGGCGACGTAAACGGCCACAAGTTCAGCGTGTCCGGCGAGGGCGAGGGCGATGCC
ACCTACGGCAAGCTGACCCTGAAGTTCATCTGCACCACCGGCAAGCTGCCCGTGCCCTGGCCCACCCTCGTGACCACCCTGACCTACGG
CGTGCAGTGCTTCAGCCGCTACCCCGACCACATGAAGCAGCACGACTTCTTCAAGTCCGCCATGCCCGAAGGCTACGTCCAGGAGCGCA
CCATCTTCTTCAAGGACGACGGCAACTACAAGACCCGCGCCGAGGTGAAGTTCGAGGGCGACACCCTGGTGAACCGCATCGAGCTGAAG
GGCATCGACTTCAAGGAGGACGGCAACATCCTGGGGCACAAGCTGGAGTACAACTACAACAGCCACAACGTCTATATCATGGCCGACAA
GCAGAAGAACGGCATCAAGGTGAACTTCAAGATCCGCCACAACATCGAGGACGGCAGCGTGCAGCTCGCCGACCACTACCAGCAGAACA
CCCCCATCGGCGACGGCCCCGTGCTGCTGCCCGACAACCACTACCTGAGCACCCAGTCCGCCCTGAGCAAAGACCCCAACGAGAAGCGC
GATCACATGGTCCTGCTGGAGTTCGTGACCGCCGCCGGGATCACTCTCGGCATGGACGAGCTGTACAAGTAAGAATTCGATATCAAGCT
TATCGATAATCAACCTCTGGATTACAAAATTTGTGAAAGATTGACTGGTATTCTTAACTATGTTGCTCCTTTTACGCTATGTGGATACG
CTGCTTTAATGCCTTTGTATCATGCTATTGCTTCCCGTATGGCTTTCATTTTCTCCTCCTTGTATAAATCCTGGTTGCTGTCTCTTTAT
GAGGAGTTGTGGCCCGTTGTCAGGCAACGTGGCGTGGTGTGCACTGTGTTTGCTGACGCAACCCCCACTGGTTGGGGCATTGCCACCAC

CTGTCAGCTCCTTTCCGGGACTTTCGCTTTCCCCCTCCCTATTGCCACGGCGGAACTCATCGCCGCCTGCCTTGCCCGCTGCTGGACAG
GGGCTCGGCTGTTGGGCACTGACAATTCCGTGGTGTTGTCGGGGAAATCATCGTCCTTTCCTTGGCTGCTCGCCTGTGTTGCCACCTGG
ATTCTGCGCGGGACGTCCTTCTGCTACGTCCCTTCGGCCCTCAATCCAGCGGACCTTCCTTCCCGCGGCCTGCTGCCGGCTCTGCGGCC
TCTTCCGCGTCTTCGCCTTCGCCCTCAGACGAGTCGGATCTCCCTTTGGGCCGCCTCCCCGCATCGATACCGTCGACCTCGAGACCTAG
AAAAACATGGAGCAATCACAAGTAGCAATACAGCAGCTACCAATGCTGATTGTGCCTGGCTAGAAGCACAAGAGGAGGAGGAGGTGGGT
TTTCCAGTCACACCTCAGGTACCTTTAAGACCAATGACTTACAAGGCAGCTGTAGATCTTAGCCACTTTTTAAAAGAAAAGGGGGGACT
GGAAGGGCTAATTCACTCCCAACGAAGACAAGATATCCTTGATCTGTGGATCTACCACACACAAGGCTACTTCCCTGATTGGCAGAACT
ACACACCAGGGCCAGGGATCAGATATCCACTGACCTTTGGATGGTGCTACAAGCTAGTACCAGTTGAGCAAGAGAAGGTAGAAGAAGCC
AATGAAGGAGAGAACACCCGCTTGTTACACCCTGTGAGCCTGCATGGGATGGATGACCCGGAGAGAGAAGTATTAGAGTGGAGGTTTGA
CAGCCGCCTAGCATTTCATCACATGGCCCGAGAGCTGCATCCGGACTGTACTGGGTCTCTCTGGTTAGACCAGATCTGAGCCTGGGAGC
TCTCTGGCTAACTAGGGAACCCACTGCTTAAGCCTCAATAAAGCTTGCCTTGAGTGCTTCAAGTAGTGTGTGCCCGTCTGTTGTGTGAC
TCTGGTAACTAGAGATCCCTCAGACCCTTTTAGTCAGTGTGGAAAATCTCTAGCAGGGCCCGTTTAAACCCGCTGATCAGCCTCGACTG
TGCCTTCTAGTTGCCAGCCATCTGTTGTTTGCCCCTCCCCCGTGCCTTCCTTGACCCTGGAAGGTGCCACTCCCACTGTCCTTTCCTAA
TAAAATGAGGAAATTGCATCGCATTGTCTGAGTAGGTGTCATTCTATTCTGGGGGGTGGGGTGGGGCAGGACAGCAAGGGGGAGGATTG
GGAAGACAATAGCAGGCATGCTGGGGATGCGGTGGGCTCTATGGCTTCTGAGGCGGAAAGAACCAGCTGGGGCTCTAGGGGGTATCCCC
ACGCGCCCTGTAGCGGCGCATTAAGCGCGGCGGGTGTGGTGGTTACGCGCAGCGTGACCGCTACACTTGCCAGCGCCCTAGCGCCCGCT
CCTTTCGCTTTCTTCCCTTCCTTTCTCGCCACGTTCGCCGGCTTTCCCCGTCAAGCTCTAAATCGGGGGCTCCCTTTAGGGTTCCGATT
TAGTGCTTTACGGCACCTCGACCCCAAAAAACTTGATTAGGGTGATGGTTCACGTAGTGGGCCATCGCCCTGATAGACGGTTTTTCGCC
CTTTGACGTTGGAGTCCACGTTCTTTAATAGTGGACTCTTGTTCCAAACTGGAACAACACTCAACCCTATCTCGGTCTATTCTTTTGAT
TTATAAGGGATTTTGCCGATTTCGGCCTATTGGTTAAAAAATGAGCTGATTTAACAAAAATTTAACGCGAATTAATTCTGTGGAATGTG
TGTCAGTTAGGGTGTGGAAAGTCCCCAGGCTCCCCAGCAGGCAGAAGTATGCAAAGCATGCATCTCAATTAGTCAGCAACCAGGTGTGG
AAAGTCCCCAGGCTCCCCAGCAGGCAGAAGTATGCAAAGCATGCATCTCAATTAGTCAGCAACCATAGTCCCGCCCCTAACTCCGCCCA
TCCCGCCCCTAACTCCGCCCAGTTCCGCCCATTCTCCGCCCCATGGCTGACTAATTTTTTTTATTTATGCAGAGGCCGAGGCCGCCTCT
GCCTCTGAGCTATTCCAGAAGTAGTGAGGAGGCTTTTTTGGAGGCCTAGGCTTTTGCAAAAAGCTCCCGGGAGCTTGTATATCCATTTT
CGGATCTGATCAGCACGTGTTGACAATTAATCATCGGCATAGTATATCGGCATAGTATAATACGACAAGGTGAGGAACTAAACCATGGC
CAAGTTGACCAGTGCCGTTCCGGTGCTCACCGCGCGCGACGTCGCCGGAGCGGTCGAGTTCTGGACCGACCGGCTCGGGTTCTCCCGGG
ACTTCGTGGAGGACGACTTCGCCGGTGTGGTCCGGGACGACGTGACCCTGTTCATCAGCGCGGTCCAGGACCAGGTGGTGCCGGACAAC
ACCCTGGCCTGGGTGTGGGTGCGCGGCCTGGACGAGCTGTACGCCGAGTGGTCGGAGGTCGTGTCCACGAACTTCCGGGACGCCTCCGG
GCCGGCCATGACCGAGATCGGCGAGCAGCCGTGGGGGCGGGAGTTCGCCCTGCGCGACCCGGCCGGCAACTGCGTGCACTTCGTGGCCG
AGGAGCAGGACTGACACGTGCTACGAGATTTCGATTCCACCGCCGCCTTCTATGAAAGGTTGGGCTTCGGAATCGTTTTCCGGGACGCC
GGCTGGATGATCCTCCAGCGCGGGGATCTCATGCTGGAGTTCTTCGCCCACCCCAACTTGTTTATTGCAGCTTATAATGGTTACAAATA
AAGCAATAGCATCACAAATTTCACAAATAAAGCATTTTTTTCACTGCATTCTAGTTGTGGTTTGTCCAAACTCATCAATGTATCTTATC
ATGTCTGTATACCGTCGACCTCTAGCTAGAGCTTGGCGTAATCATGGTCATAGCTGTTTCCTGTGTGAAATTGTTATCCGCTCACAATT
CCACACAACATACGAGCCGGAAGCATAAAGTGTAAAGCCTGGGGTGCCTAATGAGTGAGCTAACTCACATTAATTGCGTTGCGCTCACT
GCCCGCTTTCCAGTCGGGAAACCTGTCGTGCCAGCTGCATTAATGAATCGGCCAACGCGCGGGGAGAGGCGGTTTGCGTATTGGGCGCT
CTTCCGCTTCCTCGCTCACTGACTCGCTGCGCTCGGTCGTTCGGCTGCGGCGAGCGGTATCAGCTCACTCAAAGGCGGTAATACGGTTA
TCCACAGAATCAGGGGATAACGCAGGAAAGAACATGTGAGCAAAAGGCCAGCAAAAGGCCAGGAACCGTAAAAAGGCCGCGTTGCTGGC
GTTTTTCCATAGGCTCCGCCCCCCTGACGAGCATCACAAAAATCGACGCTCAAGTCAGAGGTGGCGAAACCCGACAGGACTATAAAGAT
ACCAGGCGTTTCCCCCTGGAAGCTCCCTCGTGCGCTCTCCTGTTCCGACCCTGCCGCTTACCGGATACCTGTCCGCCTTTCTCCCTTCG
GGAAGCGTGGCGCTTTCTCATAGCTCACGCTGTAGGTATCTCAGTTCGGTGTAGGTCGTTCGCTCCAAGCTGGGCTGTGTGCACGAACC
CCCCGTTCAGCCCGACCGCTGCGCCTTATCCGGTAACTATCGTCTTGAGTCCAACCCGGTAAGACACGACTTATCGCCACTGGCAGCAG
CCACTGGTAACAGGATTAGCAGAGCGAGGTATGTAGGCGGTGCTACAGAGTTCTTGAAGTGGTGGCCTAACTACGGCTACACTAGAAGA
ACAGTATTTGGTATCTGCGCTCTGCTGAAGCCAGTTACCTTCGGAAAAAGAGTTGGTAGCTCTTGATCCGGCAAACAAACCACCGCTGG
TAGCGGTGGTTTTTTTGTTTGCAAGCAGCAGATTACGCGCAGAAAAAAAGGATCTCAAGAAGATCCTTTGATCTTTTCTACGGGGTCTG
ACGCTCAGTGGAACGAAAACTCACGTTAAGGGATTTTGGTCATGAGATTATCAAAAAGGATCTTCACCTAGATCCTTTTAAATTAAAAA
TGAAGTTTTAAATCAATCTAAAGTATATATGAGTAAACTTGGTCTGACAGTTACCAATGCTTAATCAGTGAGGCACCTATCTCAGCGAT
CTGTCTATTTCGTTCATCCATAGTTGCCTGACTCCCCGTCGTGTAGATAACTACGATACGGGAGGGCTTACCATCTGGCCCCAGTGCTG
CAATGATACCGCGAGACCCACGCTCACCGGCTCCAGATTTATCAGCAATAAACCAGCCAGCCGGAAGGGCCGAGCGCAGAAGTGGTCCT
GCAACTTTATCCGCCTCCATCCAGTCTATTAATTGTTGCCGGGAAGCTAGAGTAAGTAGTTCGCCAGTTAATAGTTTGCGCAACGTTGT
TGCCATTGCTACAGGCATCGTGGTGTCACGCTCGTCGTTTGGTATGGCTTCATTCAGCTCCGGTTCCCAACGATCAAGGCGAGTTACAT
GATCCCCCATGTTGTGCAAAAAAGCGGTTAGCTCCTTCGGTCCTCCGATCGTTGTCAGAAGTAAGTTGGCCGCAGTGTTATCACTCATG
GTTATGGCAGCACTGCATAATTCTCTTACTGTCATGCCATCCGTAAGATGCTTTTCTGTGACTGGTGAGTACTCAACCAAGTCATTCTG
AGAATAGTGTATGCGGCGACCGAGTTGCTCTTGCCCGGCGTCAATACGGGATAATACCGCGCCACATAGCAGAACTTTAAAAGTGCTCA
TCATTGGAAAACGTTCTTCGGGGCGAAAACTCTCAAGGATCTTACCGCTGTTGAGATCCAGTTCGATGTAACCCACTCGTGCACCCAAC
TGATCTTCAGCATCTTTTACTTTCACCAGCGTTTCTGGGTGAGCAAAAACAGGAAGGCAAAATGCCGCAAAAAAGGGAATAAGGGCGAC
ACGGAAATGTTGAATACTCATACTCTTCCTTTTTCAATATTATTGAAGCATTTATCAGGGTTATTGTCTCATGAGCGGATACATATTTG
AATGTATTTAGAAAAATAAACAAATAGGGGTTCCGCGCACATTTCCCCGAAAAGTGCCACCTGAC

Lentiviral plasmid DNA reconstruction: Whole plasmid sequence 2

(ii) eGFP-intron splicing system

Vector name : Forward-EF1a-eGFP-intron / FUGW

Feature Name
CMV_immearly_promoter / HIV-1_5_LTR / truncHIV-1_3_LTR / HIV-1_psi_pack / RRE / cPPT / EF1a_promoter / eGFP-intron / WPRE / U3PPT / HIV-1_5_LTR

GTCGACGGATCGGGAGATCTCCCGATCCCCTATGGTGCACTCTCAGTACAATCTGCTCTGATGCCGCATAGTTAAGCCAGTATCTGCTC
CCTGCTTGTGTGTTGGAGGTCGCTGAGTAGTGCGCGAGCAAAATTTAAGCTACAACAAGGCAAGGCTTGACCGACAATTGCATGAAGAA
TCTGCTTAGGGTTAGGCGTTTTGCGCTGCTTCGCGATGTACGGGCCAGATATACGCGTTGACATTGATTATTGACTAGTTATTAATAGT
AATCAATTACGGGGTCATTAGTTCATAGCCCATATATGGAGTTCCGCGTTACATAACTTACGGTAAATGGCCCGCCTGGCTGACCGCCC
AACGACCCCCGCCCATTGACGTCAATAATGACGTATGTTCCCATAGTAACGCCAATAGGGACTTTCCATTGACGTCAATGGGTGGAGTA
TTTACGGTAAACTGCCCACTTGGCAGTACATCAAGTGTATCATATGCCAAGTACGCCCCCTATTGACGTCAATGACGGTAAATGGCCCG
CCTGGCATTATGCCCAGTACATGACCTTATGGGACTTTCCTACTTGGCAGTACATCTACGTATTAGTCATCGCTATTACCATGGTGATG
CGGTTTTGGCAGTACATCAATGGGCGTGGATAGCGGTTTGACTCACGGGGATTTCCAAGTCTCCACCCCATTGACGTCAATGGGAGTTT
GTTTTGGCACCAAAATCAACGGGACTTTCCAAAATGTCGTAACAACTCCGCCCCATTGACGCAAATGGGCGGTAGGCGTGTACGGTGGG
AGGTC**TATATAA**GCAGCGCGTTTTGCCTGTACTGGGTCTCTCTGGTTAGACCAGATCTGAGCCTGGGAGCTCTCTGGCTAACTAGGGAA
CCCACTGCTTAAGCCTCAATAAAGCTTGCCTTGAGTGCTTCAAGTAGTGTGTGCCCGTCTGTTGTGTGACTCTGGTAACTAGAGATCCC
TCAGACCCTTTTAGTCAGTGTGGAAAATCTCTAGCAGTGGCGCCCGAACAGGGACTTGAAAGCGAAAGGGAAACCAGAGGAGCTCTCTC
GACGCAGGACTCGGCTTGCTGAAGCGCGCACGGCAAGAGGCGAGGGGCGGCGACTGGTGAGTACGCCAAAAATTTTGACTAGCGGAGGC
TAGAAGGAGAGAGATGGGTGCGAGAGCGTCAGTATTAAGCGGGGGAGAATTAGATCGCGATGGGAAAAAATTCGGTTAAGGCCAGGGGG
AAAGAAAAAATATAAATTAAAACATATAGTATGGGCAAGCAGGGAGCTAGAACGATTCGCAGTTAATCCTGGCCTGTTAGAAACATCAG
AAGGCTGTAGACAAATACTGGGACAGCTACAACCATCCCTTCAGACAGGATCAGAAGAACTTAGATCATTATATAATACAGTAGCAACC
CTCTATTGTGTGCATCAAAGGATAGAGATAAAAGACACCAAGGAAGCTTTAGACAAGATAGAGGAAGAGCAAAACAAAAGTAAGACCAC
CGCACAGCAAGCGGCCGCTGATCTTCAGACCTGGAGGAGGAGATATGAGGGACAATTGGAGAAGTGAATTATATAAATATAAAGTAGTA
AAAATTGAACCATTAGGAGTAGCACCCACCAAGGCAAAGAGAAGAGTGGTGCAGAGAGAAAAAAGAGCAGTGGGAATAGGAGCTTTGTT
CCTTGGGTTCTTGGGAGCAGCAGGAAGCACTATGGGCGCAGCGTCAATGACGCTGACGGTACAGGCCAGACAATTATTGTCTGGTATAG
TGCAGCAGCAGAACAATTTGCTGAGGGCTATTGAGGCGCAACAGCATCTGTTGCAACTCACAGTCTGGGGCATCAAGCAGCTCCAGGCA
AGAATCCTGGCTGTGGAAAGATACCTAAAGGATCAACAGCTCCTGGGGATTTGGGGTTGCTCTGGAAAACTCATTTGCACCACTGCTGT
GCCTTGGAATGCTAGTTGGAGTAATAAATCTCTGGAACAGATTTGGAATCACACGACCTGGATGGAGTGGGACAGAGAAATTAACAATT
ACACAAGCTTAATACACTCCTTAATTGAAGAATCGCAAAACCAGCAAGAAAAGAATGAACAAGAATTATTGGAATTAGATAAATGGGCA
AGTTTGTGGAATTGGTTTAACATAACAAATTGGCTGTGGTATATAAAATTATTCATAATGATAGTAGGAGGCTTGGTAGGTTTAAGAAT
AGTTTTTGCTGTACTTTCTATAGTGAATAGAGTTAGGCAGGGATATTCACCATTATCGTTTCAGACCCACCTCCCAACCCCGAGGGGAC
CCGACAGGCCCGAAGGAATAGAAGAAGAAGGTGGAGAGAGAGACAGAGACAGATCCATTCGATTAGTGAACGGATCGGCACTGCGTGCG
CCAATTCTGCAGACAAATGGCAGTATTCATCCACAATTTTAAAAGAAAAGGGGGGATTGGGGGGTACAGTGCAGGGGAAAGAATAGTAG
ACATAATAGCAACAGACATACAAACTAAAGAATTACAAAAACAAATTACAAAAATTCAAAATTTTCGGGTTTATTACAGGGACAGCAGA
GATCCAGTTTGGttaattaacgtgaggctccggtgcccgtcagtgggcagagcgcacatcgcccacagtccccgagaagttggggggag
gggtcggcaattgaaccggtgcctagagaaggtggcgcggggtaaactgggaaagtgatgtcgtgtactggctccgcctttttcccgag
ggtgggggagaaccgtatataagtgcagtagtcgccgtgaacgttctttttcgcaacgggtttgccgccagaacacaggtaagtgccgt
gtgtggttcccgcgggcctggcctctttacgggttatggcccttgcgtgccttgaattacttccacgcccctggctgcagtacgtgatt
cttgatcccgagcttcgggttggaagtgggtgggagagttcgaggccttgcgcttaaggagccccttcgcctcgtgcttgagttgaggc
ctggcctgggcgctggggccgccgcgtgcgaatctggtggcaccttcgcgcctgtctcgctgctttcgataagtctctagccatttaaa
atttttgatgacctgctgcgacgctttttttctggcaagatagtcttgtaaatgcgggccaagatctgcacactggtatttcggttttt
ggggccgcgggcggcgacggggcccgtgcgtcccagcgcacatgttcggcgaggcggggcctgcgagcgcggccaccgagaatcggacg
gggggtagtctcaagctggccggcctgctctggtgcctggcctcgcgccgccgtgtatcgccccgccctgggcggcaaggctggcccggt
cggcaccagttgcgtgagcggaaagatggccgcttcccggccctgctgcagggagctcaaaatggaggacgcggcgctcgggagagcgg
gcgggtgagtcacccacacaaaggaaaagggcctttccgtcctcagccgtcgcttcatgtgactccacggagtaccgggcgccgtccag
gcacctcgattagttctcgagcttttggagtacgtcgtctttaggttggggggaggggttttatgcgatggagtttccccacactgagt
gggtggagactgaagttaggccagcttggcacttgatgtaattctccttggaatttgccctttttgagtttggatcttggttcattc**tc**
aagcctcagacagtggttcaaagtttttttcttccatttcaggtgtcgtgagggatccGCCACCATGGTGAGCAAGGGCGAGGAGCTGT
TCACCGGGGTGGTGCCCATCCTGGTCGAGCTGGACGGCGACGTAAACGGCCACAAGTTCAGCGTGTCCGGCGAGGGCGAGGGCGATGCC
ACCTACGGCAAGCTGACCCTGAAGTTCATCTGCACCACCGGCAAGCTGCCCGTGCCCTGGCCCACCCTCGTGACCACCCTGACCTACGG
CGTGCAGTGCTTCAGCCGCTACCCCGACCACATGAAGCAGCACGACTTCTTCAAGTCCGCCATGCCCGAAGGCTACGTCCAGGAGCGCA
CCATCTTCTTCAAGGACGACGGCAACTACAAGACCCGCGCCGAGGTAAGTATCAAGGTTACAAGACAGGTTTAAGGAGACCAATAGAAA
CTGGGCTTGTCGAGACAGAGAAGACTCTTGCGTTTTCGAT**GAGACG**TACAGATG**CGTCTC**AGGCCATGATAGGCACCTATTGGTCTTA**C**
TGACATCCACTTTGCCTTTCTCTCCACAGGTGAAGTTCGAGGGCGACACCCTGGTGAACCGCATCGAGCTGAAGGGCATCGACTTCAAG
GAGGACGGCAACATCCTGGGGCACAAGCTGGAGTACAACTACAACAGCCACAACGTCTATATCATGGCCGACAAGCAGAAGAACGGCAT
CAAGGTGAACTTCAAGATCCGCCACAACATCGAGGACGGCAGCGTGCAGCTCGCCGACCACTACCAGCAGAACACCCCCATCGGCGACG
GCCCCGTGCTGCTGCCCGACAACCACTACCTGAGCACCCAGTCCGCCCTGAGCAAAGACCCCAACGAGAAGCGCGATCACATGGTCCTG
CTGGAGTTCGTGACCGCCGCCGGGATCACTCTCGGCATGGACGAGCTGTACAAGTAAGAATTCGATATCAAGCTTATCGATAATCAACC
TCTGGATTACAAAATTTGTGAAAGATTGACTGGTATTCTTAACTATGTTGCTCCTTTTACGCTATGTGGATACGCTGCTTTAATGCCTT

TGTATCATGCTATTGCTTCCCGTATGGCTTTCATTTTCTCCTCCTTGTATAAATCCTGGTTGCTGTCTCTTTATGAGGAGTTGTGGCCC
GTTGTCAGGCAACGTGGCGTGGTGTGCACTGTGTTTGCTGACGCAACCCCCACTGGTTGGGGCATTGCCACCACCTGTCAGCTCCTTTC
CGGGACTTTCGCTTTCCCCCTCCCTATTGCCACGGCGGAACTCATCGCCGCCTGCCTTGCCCGCTGCTGGACAGGGGCTCGGCTGTTGG
GCACTGACAATTCCGTGGTGTTGTCGGGGAAATCATCGTCCTTTCCTTGGCTGCTCGCCTGTGTTGCCACCTGGATTCTGCGCGGGACG
TCCTTCTGCTACGTCCCTTCGGCCCTCAATCCAGCGGACCTTCCTTCCCGCGGCCTGCTGCCGGCTCTGCGGCCTCTTCCGCGTCTTCG
CCTTCGCCCTCAGACGAGTCGGATCTCCCTTTGGGCCGCCTCCCCGCATCGATACCGTCGACCTCGAGACCTAGAAAAACATGGAGCAA
TCACAAGTAGCAATACAGCAGCTACCAATGCTGATTGTGCCTGGCTAGAAGCACAAGAGGAGGAGGAGGTGGGTTTTCCAGTCACACCT
CAGGTACCTTTAAGACCAATGACTTACAAGGCAGCTGTAGATCTTAGCCACTTTTTAAAAGAAAAGGGGGGACTGGAAGGGCTAATTCA
CTCCCAACGAAGACAAGATATCCTTGATCTGTGGATCTACCACACACAAGGCTACTTCCCTGATTGGCAGAACTACACACCAGGGCCAG
GGATCAGATATCCACTGACCTTTGGATGGTGCTACAAGCTAGTACCAGTTGAGCAAGAGAAGGTAGAAGAAGCCAATGAAGGAGAGAAC
ACCCGCTTGTTACACCCTGTGAGCCTGCATGGGATGGATGACCCGGAGAGAGAAGTATTAGAGTGGAGGTTTGACAGCCGCCTAGCATT
TCATCACATGGCCCGAGAGCTGCATCCGGACTGTACTGGGTCTCTCTGGTTAGACCAGATCTGAGCCTGGGAGCTCTCTGGCTAACTAG
GGAACCCACTGCTTAAGCCTCAATAAAGCTTGCCTTGAGTGCTTCAAGTAGTGTGTGCCCGTCTGTTGTGTGACTCTGGTAACTAGAGA
TCCCTCAGACCCTTTTAGTCAGTGTGGAAAATCTCTAGCAGGGCCCGTTTAAACCCGCTGATCAGCCTCGACTGTGCCTTCTAGTTGCC
AGCCATCTGTTGTTTGCCCCTCCCCCGTGCCTTCCTTGACCCTGGAAGGTGCCACTCCCACTGTCCTTTCCTAATAAAATGAGGAAATT
GCATCGCATTGTCTGAGTAGGTGTCATTCTATTCTGGGGGGTGGGGTGGGGCAGGACAGCAAGGGGGAGGATTGGGAAGACAATAGCAG
GCATGCTGGGGATGCGGTGGGCTCTATGGCTTCTGAGGCGGAAAGAACCAGCTGGGGCTCTAGGGGGTATCCCCACGCGCCCTGTAGCG
GCGCATTAAGCGCGGCGGGTGTGGTGGTTACGCGCAGCGTGACCGCTACACTTGCCAGCGCCCTAGCGCCCGCTCCTTTCGCTTTCTTC
CCTTCCTTTCTCGCCACGTTCGCCGGCTTTCCCCGTCAAGCTCTAAATCGGGGGCTCCCTTTAGGGTTCCGATTTAGTGCTTTACGGCA
CCTCGACCCCAAAAAACTTGATTAGGGTGATGGTTCACGTAGTGGGCCATCGCCCTGATAGACGGTTTTTCGCCCTTTGACGTTGGAGT
CCACGTTCTTTAATAGTGGACTCTTGTTCCAAACTGGAACAACACTCAACCCTATCTCGGTCTATTCTTTTGATTTATAAGGGATTTTG
CCGATTTCGGCCTATTGGTTAAAAAATGAGCTGATTTAACAAAAATTTAACGCGAATTAATTCTGTGGAATGTGTGTCAGTTAGGGTGT
GGAAAGTCCCCAGGCTCCCCAGCAGGCAGAAGTATGCAAAGCATGCATCTCAATTAGTCAGCAACCAGGTGTGGAAAGTCCCCAGGCTC
CCCAGCAGGCAGAAGTATGCAAAGCATGCATCTCAATTAGTCAGCAACCATAGTCCCGCCCCTAACTCCGCCCATCCCGCCCCTAACTC
CGCCCAGTTCCGCCCATTCTCCGCCCCATGGCTGACTAATTTTTTTTATTTATGCAGAGGCCGAGGCCGCCTCTGCCTCTGAGCTATTC
CAGAAGTAGTGAGGAGGCTTTTTTGGAGGCCTAGGCTTTTGCAAAAAGCTCCCGGGAGCTTGTATATCCATTTTCGGATCTGATCAGCA
CGTGTTGACAATTAATCATCGGCATAGTATATCGGCATAGTATAATACGACAAGGTGAGGAACTAAACCATGGCCAAGTTGACCAGTGC
CGTTCCGGTGCTCACCGCGCGCGACGTCGCCGGAGCGGTCGAGTTCTGGACCGACCGGCTCGGGTTCTCCCGGGACTTCGTGGAGGACG
ACTTCGCCGGTGTGGTCCGGGACGACGTGACCCTGTTCATCAGCGCGGTCCAGGACCAGGTGGTGCCGGACAACACCCTGGCCTGGGTG
TGGGTGCGCGGCCTGGACGAGCTGTACGCCGAGTGGTCGGAGGTCGTGTCCACGAACTTCCGGGACGCCTCCGGGCCGGCCATGACCGA
GATCGGCGAGCAGCCGTGGGGGCGGGAGTTCGCCCTGCGCGACCCGGCCGGCAACTGCGTGCACTTCGTGGCCGAGGAGCAGGACTGAC
ACGTGCTACGAGATTTCGATTCCACCGCCGCCTTCTATGAAAGGTTGGGCTTCGGAATCGTTTTCCGGGACGCCGGCTGGATGATCCTC
CAGCGCGGGGATCTCATGCTGGAGTTCTTCGCCCACCCCAACTTGTTTATTGCAGCTTATAATGGTTACAAATAAAGCAATAGCATCAC
AAATTTCACAAATAAAGCATTTTTTTCACTGCATTCTAGTTGTGGTTTGTCCAAACTCATCAATGTATCTTATCATGTCTGTATACCGT
CGACCTCTAGCTAGAGCTTGGCGTAATCATGGTCATAGCTGTTTCCTGTGTGAAATTGTTATCCGCTCACAATTCCACACAACATACGA
GCCGGAAGCATAAAGTGTAAAGCCTGGGGTGCCTAATGAGTGAGCTAACTCACATTAATTGCGTTGCGCTCACTGCCCGCTTTCCAGTC
GGGAAACCTGTCGTGCCAGCTGCATTAATGAATCGGCCAACGCGCGGGGAGAGGCGGTTTGCGTATTGGGCGCTCTTCCGCTTCCTCGC
TCACTGACTCGCTGCGCTCGGTCGTTCGGCTGCGGCGAGCGGTATCAGCTCACTCAAAGGCGGTAATACGGTTATCCACAGAATCAGGG
GATAACGCAGGAAAGAACATGTGAGCAAAAGGCCAGCAAAAGGCCAGGAACCGTAAAAAGGCCGCGTTGCTGGCGTTTTTCCATAGGCT
CCGCCCCCCTGACGAGCATCACAAAAATCGACGCTCAAGTCAGAGGTGGCGAAACCCGACAGGACTATAAAGATACCAGGCGTTTCCCC
CTGGAAGCTCCCTCGTGCGCTCTCCTGTTCCGACCCTGCCGCTTACCGGATACCTGTCCGCCTTTCTCCCTTCGGGAAGCGTGGCGCTT
TCTCATAGCTCACGCTGTAGGTATCTCAGTTCGGTGTAGGTCGTTCGCTCCAAGCTGGGCTGTGTGCACGAACCCCCCGTTCAGCCCGA
CCGCTGCGCCTTATCCGGTAACTATCGTCTTGAGTCCAACCCGGTAAGACACGACTTATCGCCACTGGCAGCAGCCACTGGTAACAGGA
TTAGCAGAGCGAGGTATGTAGGCGGTGCTACAGAGTTCTTGAAGTGGTGGCCTAACTACGGCTACACTAGAAGAACAGTATTTGGTATC
TGCGCTCTGCTGAAGCCAGTTACCTTCGGAAAAAGAGTTGGTAGCTCTTGATCCGGCAAACAAACCACCGCTGGTAGCGGTGGTTTTTT
TGTTTGCAAGCAGCAGATTACGCGCAGAAAAAAAGGATCTCAAGAAGATCCTTTGATCTTTTCTACGGGGTCTGACGCTCAGTGGAACG
AAAACTCACGTTAAGGGATTTTGGTCATGAGATTATCAAAAAGGATCTTCACCTAGATCCTTTTAAATTAAAAATGAAGTTTTAAATCA
ATCTAAAGTATATATGAGTAAACTTGGTCTGACAGTTACCAATGCTTAATCAGTGAGGCACCTATCTCAGCGATCTGTCTATTTCGTTC
ATCCATAGTTGCCTGACTCCCCGTCGTGTAGATAACTACGATACGGGAGGGCTTACCATCTGGCCCCAGTGCTGCAATGATACCGCGAG
ACCCACGCTCACCGGCTCCAGATTTATCAGCAATAAACCAGCCAGCCGGAAGGGCCGAGCGCAGAAGTGGTCCTGCAACTTTATCCGCC
TCCATCCAGTCTATTAATTGTTGCCGGGAAGCTAGAGTAAGTAGTTCGCCAGTTAATAGTTTGCGCAACGTTGTTGCCATTGCTACAGG
CATCGTGGTGTCACGCTCGTCGTTTGGTATGGCTTCATTCAGCTCCGGTTCCCAACGATCAAGGCGAGTTACATGATCCCCCATGTTGT
GCAAAAAAGCGGTTAGCTCCTTCGGTCCTCCGATCGTTGTCAGAAGTAAGTTGGCCGCAGTGTTATCACTCATGGTTATGGCAGCACTG
CATAATTCTCTTACTGTCATGCCATCCGTAAGATGCTTTTCTGTGACTGGTGAGTACTCAACCAAGTCATTCTGAGAATAGTGTATGCG
GCGACCGAGTTGCTCTTGCCCGGCGTCAATACGGGATAATACCGCGCCACATAGCAGAACTTTAAAAGTGCTCATCATTGGAAAACGTT
CTTCGGGGCGAAAACTCTCAAGGATCTTACCGCTGTTGAGATCCAGTTCGATGTAACCCACTCGTGCACCCAACTGATCTTCAGCATCT
TTTACTTTCACCAGCGTTTCTGGGTGAGCAAAAACAGGAAGGCAAAATGCCGCAAAAAAGGGAATAAGGGCGACACGGAAATGTTGAAT
ACTCATACTCTTCCTTTTTCAATATTATTGAAGCATTTATCAGGGTTATTGTCTCATGAGCGGATACATATTTGAATGTATTTAGAAAA
ATAAACAAATAGGGGTTCCGCGCACATTTCCCCGAAAAGTGCCACCTGAC

Lentiviral plasmid DNA reconstruction: Whole plasmid sequence 3

(iv) Forward vector / Reverse vector

Vector name: Reverse-EF1a-eGFP-intron / FUGW

Feature Name
CMV_immearly_promoter / HIV-1_5_LTR / truncHIV-1_3_LTR / HIV-1_psi_pack / RRE / cPPT / SV40 polyadenylation signal (Reverse) / WPRE (Reverse) / eGFP-intron (Reverse) / EF1a_promoter (Reverse) / U3PPT / HIV-1_5_LTR

GTCGACGGATCGGGAGATCTCCCGATCCCCTATGGTGCACTCTCAGTACAATCTGCTCTGATGCCGCATAGTTAAGCCAGTATCTGCTC
CCTGCTTGTGTGTTGGAGGTCGCTGAGTAGTGCGCGAGCAAAATTTAAGCTACAACAAGGCAAGGCTTGACCGACAATTGCATGAAGAA
TCTGCTTAGGGTTAGGCGTTTTGCGCTGCTTCGCGATGTACGGGCCAGATATACGCGTTGACATTGATTATTGACTAGTTATTAATAGT
AATCAATTACGGGGTCATTAGTTCATAGCCCATATATGGAGTTCCGCGTTACATAACTTACGGTAAATGGCCCGCCTGGCTGACCGCCC
AACGACCCCCGCCCATTGACGTCAATAATGACGTATGTTCCCATAGTAACGCCAATAGGGACTTTCCATTGACGTCAATGGGTGGAGTA
TTTACGGTAAACTGCCCACTTGGCAGTACATCAAGTGTATCATATGCCAAGTACGCCCCCTATTGACGTCAATGACGGTAAATGGCCCG
CCTGGCATTATGCCCAGTACATGACCTTATGGGACTTTCCTACTTGGCAGTACATCTACGTATTAGTCATCGCTATTACCATGGTGATG
CGGTTTTGGCAGTACATCAATGGGCGTGGATAGCGGTTTGACTCACGGGGATTTCCAAGTCTCCACCCCATTGACGTCAATGGGAGTTT
GTTTTGGCACCAAAATCAACGGGACTTTCCAAAATGTCGTAACAACTCCGCCCCATTGACGCAAATGGGCGGTAGGCGTGTACGGTGGG
AGGTC**TATATAA**GCAGCGCGTTTTGCCTGTACTGGGTCTCTCTGGTTAGACCAGATCTGAGCCTGGGAGCTCTCTGGCTAACTAGGGAA
CCCACTGCTTAAGCCTCAATAAAGCTTGCCTTGAGTGCTTCAAGTAGTGTGTGCCCGTCTGTTGTGTGACTCTGGTAACTAGAGATCCC
TCAGACCCTTTTAGTCAGTGTGGAAAATCTCTAGCAGTGGCGCCCGAACAGGGACTTGAAAGCGAAAGGGAAACCAGAGGAGCTCTCTC
GACGCAGGACTCGGCTTGCTGAAGCGCGCACGGCAAGAGGCGAGGGGCGGCGACTGGTGAGTACGCCAAAAATTTTGACTAGCGGAGGC
TAGAAGGAGAGAGATGGGTGCGAGAGCGTCAGTATTAAGCGGGGGAGAATTAGATCGCGATGGGAAAAAATTCGGTTAAGGCCAGGGGG
AAAGAAAAAATATAAATTAAAACATATAGTATGGGCAAGCAGGGAGCTAGAACGATTCGCAGTTAATCCTGGCCTGTTAGAAACATCAG
AAGGCTGTAGACAAATACTGGGACAGCTACAACCATCCCTTCAGACAGGATCAGAAGAACTTAGATCATTATATAATACAGTAGCAACC
CTCTATTGTGTGCATCAAAGGATAGAGATAAAAGACACCAAGGAAGCTTTAGACAAGATAGAGGAAGAGCAAAACAAAAGTAAGACCAC
CGCACAGCAAGCGGCCGCTGATCTTCAGACCTGGAGGAGGAGATATGAGGGACAATTGGAGAAGTGAATTATATAAATATAAAGTAGTA
AAAATTGAACCATTAGGAGTAGCACCCACCAAGGCAAAGAGAAGAGTGGTGCAGAGAGAAAAAAGAGCAGTGGGAATAGGAGCTTTGTT
CCTTGGGTTCTTGGGAGCAGCAGGAAGCACTATGGGCGCAGCGTCAATGACGCTGACGGTACAGGCCAGACAATTATTGTCTGGTATAG
TGCAGCAGCAGAACAATTTGCTGAGGGCTATTGAGGCGCAACAGCATCTGTTGCAACTCACAGTCTGGGGCATCAAGCAGCTCCAGGCA
AGAATCCTGGCTGTGGAAAGATACCTAAAGGATCAACAGCTCCTGGGGATTTGGGGTTGCTCTGGAAAACTCATTTGCACCACTGCTGT
GCCTTGGAATGCTAGTTGGAGTAATAAATCTCTGGAACAGATTTGGAATCACACGACCTGGATGGAGTGGGACAGAGAAATTAACAATT
ACACAAGCTTAATACACTCCTTAATTGAAGAATCGCAAAACCAGCAAGAAAAGAATGAACAAGAATTATTGGAATTAGATAAATGGGCA
AGTTTGTGGAATTGGTTTAACATAACAAATTGGCTGTGGTATATAAAATTATTCATAATGATAGTAGGAGGCTTGGTAGGTTTAAGAAT
AGTTTTTGCTGTACTTTCTATAGTGAATAGAGTTAGGCAGGGATATTCACCATTATCGTTTCAGACCCACCTCCCAACCCCGAGGGGAC
CCGACAGGCCCGAAGGAATAGAAGAAGAAGGTGGAGAGAGAGACAGAGACAGATCCATTCGATTAGTGAACGGATCGGCACTGCGTGCG
CCAATTCTGCAGACAAATGGCAGTATTCATCCACAATTTTAAAAGAAAAGGGGGGATTGGGGGGTACAGTGCAGGGGAAAGAATAGTAG
ACATAATAGCAACAGACATACAAACTAAAGAATTACAAAAACAAATTACAAAAATTCAAAATTTTCGGGTTTATTACAGGGACAGCAGA
GATCCAGTTTGGttaatacaaaccacaactagaatgcagtgaaaaaaatgctttattGGTACCTGAGGTGTGACTGGAAAACCCACCTC
CTCCTCCTCTTGTGCTTCTAGCCAGGCACAATCAGCATTGGTAGCTGCTGTATTGCTACTTGTGATTGCTCCATGTTTTTCTAGGTCTC
GAGGTCGACGGTATCGATGCGGGGAGGCGGCCCAAAGGGAGATCCGACTCGTCTGAGGGCGAAGGCGAAGACGCGGAAGAGGCCGCAGA
GCCGGCAGCAGGCCGCGGGAAGGAAGGTCCGCTGGATTGAGGGCCGAAGGGACGTAGCAGAAGGACGTCCCGCGCAGAATCCAGGTGGC
AACACAGGCGAGCAGCCAAGGAAAGGACGATGATTTCCCCGACAACACCACGGAATTGTCAGTGCCCAACAGCCGAGCCCCTGTCCAGC
AGCGGGCAAGGCAGGCGGCGATGAGTTCCGCCGTGGCAATAGGGAGGGGGAAAGCGAAAGTCCCGGAAAGGAGCTGACAGGTGGTGGCA
ATGCCCCAACCAGTGGGGGTTGCGTCAGCAAACACAGTGCACACCACGCCACGTTGCCTGACAACGGGCCACAACTCCTCATAAAGAGA
CAGCAACCAGGATTTATACAAGGAGGAGAAAATGAAAGCCATACGGGAAGCAATAGCATGATACAAAGGCATTAAAGCAGCGTATCCAC
ATAGCGTAAAAGGAGCAACATAGTTAAGAATACCAGTCAATCTTTCACAAATTTTGTAATCCAGAGGTTGATTATCGATAAGCTTGATA
TCGAATTCTTACTTGTACAGCTCGTCCATGCCGAGAGTGATCCCGGCGGCGGTCACGAACTCCAGCAGGACCATGTGATCGCGCTTCTC
GTTGGGGTCTTTGCTCAGGGCGGACTGGGTGCTCAGGTAGTGGTTGTCGGGCAGCAGCACGGGGCCGTCGCCGATGGGGGTGTTCTGCT
GGTAGTGGTCGGCGAGCTGCACGCTGCCGTCCTCGATGTTGTGGCGGATCTTGAAGTTCACCTTGATGCCGTTCTTCTGCTTGTCGGCC
ATGATATAGACGTTGTGGCTGTTGTAGTTGTACTCCAGCTTGTGCCCCAGGATGTTGCCGTCCTCCTTGAAGTCGATGCCCTTCAGCTC
GATGCGGTTCACCAGGGTGTCGCCCTCGAACTTCACCTGTGGAGAGAAAGGCAAAGTGGATGTCAGTAAGACCAATAGGTGCCTATCAT
GGCCTGAGACGCATCTGTACGTCTCATCGAAAACGCAAGAGTCTTCTCTGTCTCGACAAGCCCAGTTTCTATTGGTCTCCTTAAACCTG
TCTTGTAACCTTGATACTTACCTCGGCGCGGGTCTTGTAGTTGCCGTCGTCCTTGAAGAAGATGGTGCGCTCCTGGACGTAGCCTTCGG
GCATGGCGGACTTGAAGAAGTCGTGCTGCTTCATGTGGTCGGGGTAGCGGCTGAAGCACTGCACGCCGTAGGTCAGGGTGGTCACGAGG
GTGGGCCAGGGCACGGGCAGCTTGCCGGTGGTGCAGATGAACTTCAGGGTCAGCTTGCCGTAGGTGGCATCGCCCTCGCCCTCGCCGGA
CACGCTGAACTTGTGGCCGTTTACGTCGCCGTCCAGCTCGACCAGGATGGGCACCACCCCGGTGAACAGCTCCTCGCCCTTGCTCACCA
TGGTGGCggatccCTCACGACACCTGAAATGGAAGAAAAAAACTTTGAACCACTGTCTGAGGCTTGAGAATGAACCAAGATCCAAACTC
AAAAAGGGCAAATTCCAAGGAGAATTACATCAAGTGCCAAGCTGGCCTAACTTCAGTCTCCACCCACTCAGTGTGGGGAAACTCCATCG
CATAAAACCCCTCCCCCCAACCTAAAGACGACGTACTCCAAAAGCTCGAGAACTAATCGAGGTGCCTGGACGGCGCCCGGTACTCCGTG
GAGTCACATGAAGCGACGGCTGAGGACGGAAAGGCCCTTTTCCTTTGTGTGGGTGACTCACCCGCCCGCTCTCCCGAGCGCCGCGTCCT
CCATTTTGAGCTCCCTGCAGCAGGGCCGGGAAGCGGCCATCTTTCCGCTCACGCAACTGGTGCCGACCGGGCCAGCCTTGCCGCCCAGG

GCGGGGCGATACACGGCGGCGCGAGGCCAGGCACCAGAGCAGGCCGGCCAGCTTGAGACTACCCCCGTCCGATTCTCGGTGGCCGCGCT
CGCAGGCCCCGCCTCGCCGAACATGTGCGCTGGGACGCACGGGCCCCGTCGCCGCCCGCGGCCCCAAAAACCGAAATACCAGTGTGCAG
ATCTTGGCCCGCATTTACAAGACTATCTTGCCAGAAAAAAAGCGTCGCAGCAGGTCATCAAAAATTTTAAATGGCTAGAGACTTATCGA
AAGCAGCGAGACAGGCGCGAAGGTGCCACCAGATTCGCACGCGGCGGCCCCAGCGCCCAGGCCAGGCCTCAACTCAAGCACGAGGCGAA
GGGGCTCCTTAAGCGCAAGGCCTCGAACTCTCCCACCCACTTCCAACCCGAAGCTCGGGATCAAGAATCACGTACTGCAGCCAGGGGCG
TGGAAGTAATTCAAGGCACGCAAGGGCCATAACCCGTAAAGAGGCCAGGCCCGCGGGAACCACACACGGCACTTACCTGTGTTCTGGCG
GCAAACCCGTTGCGAAAAAGAACGTTCACGGCGACTACTGCACTTATATACGGTTCTCCCCCACCCTCGGGAAAAAGGCGGAGCCAGTA
CACGACATCACTTTCCCAGTTTACCCCGCGCCACCTTCTCTAGGCACCGGTTCAATTGCCGACCCCTCCCCCCAACTTCTCGGGGACTG
TGGGCGATGTGCGCTCTGCCCACTGACGGGCACCGGAGCCTCACGTTAATTAAGTACCTTTAAGACCAATGACTTACAAGGCAGCTGTA
GATCTTAGCCACTTTTTAAAAGAAAAGGGGGGACTGGAAGGGCTAATTCACTCCCAACGAAGACAAGATATCCTTGATCTGTGGATCTA
CCACACACAAGGCTACTTCCCTGATTGGCAGAACTACACACCAGGGCCAGGGATCAGATATCCACTGACCTTTGGATGGTGCTACAAGC
TAGTACCAGTTGAGCAAGAGAAGGTAGAAGAAGCCAATGAAGGAGAGAACACCCGCTTGTTACACCCTGTGAGCCTGCATGGGATGGAT
GACCCGGAGAGAGAAGTATTAGAGTGGAGGTTTGACAGCCGCCTAGCATTTCATCACATGGCCCGAGAGCTGCATCCGGACTGTACTGG
GTCTCTCTGGTTAGACCAGATCTGAGCCTGGGAGCTCTCTGGCTAACTAGGGAACCCACTGCTTAAGCCTCAATAAAGCTTGCCTTGAG
TGCTTCAAGTAGTGTGTGCCCGTCTGTTGTGTGACTCTGGTAACTAGAGATCCCTCAGACCCTTTTAGTCAGTGTGGAAAATCTCTAGC
AGGGCCCGTTTAAACCCGCTGATCAGCCTCGACTGTGCCTTCTAGTTGCCAGCCATCTGTTGTTTGCCCCTCCCCCGTGCCTTCCTTGA
CCCTGGAAGGTGCCACTCCCACTGTCCTTTCCTAATAAAATGAGGAAATTGCATCGCATTGTCTGAGTAGGTGTCATTCTATTCTGGGG
GGTGGGGTGGGGCAGGACAGCAAGGGGGAGGATTGGGAAGACAATAGCAGGCATGCTGGGGATGCGGTGGGCTCTATGGCTTCTGAGGC
GGAAAGAACCAGCTGGGGCTCTAGGGGGTATCCCCACGCGCCCTGTAGCGGCGCATTAAGCGCGGCGGGTGTGGTGGTTACGCGCAGCG
TGACCGCTACACTTGCCAGCGCCCTAGCGCCCGCTCCTTTCGCTTTCTTCCCTTCCTTTCTCGCCACGTTCGCCGGCTTTCCCCGTCAA
GCTCTAAATCGGGGGCTCCCTTTAGGGTTCCGATTTAGTGCTTTACGGCACCTCGACCCCAAAAAACTTGATTAGGGTGATGGTTCACG
TAGTGGGCCATCGCCCTGATAGACGGTTTTTCGCCCTTTGACGTTGGAGTCCACGTTCTTTAATAGTGGACTCTTGTTCCAAACTGGAA
CAACACTCAACCCTATCTCGGTCTATTCTTTTGATTTATAAGGGATTTTGCCGATTTCGGCCTATTGGTTAAAAAATGAGCTGATTTAA
CAAAAATTTAACGCGAATTAATTCTGTGGAATGTGTGTCAGTTAGGGTGTGGAAAGTCCCCAGGCTCCCCAGCAGGCAGAAGTATGCAA
AGCATGCATCTCAATTAGTCAGCAACCAGGTGTGGAAAGTCCCCAGGCTCCCCAGCAGGCAGAAGTATGCAAAGCATGCATCTCAATTA
GTCAGCAACCATAGTCCCGCCCCTAACTCCGCCCATCCCGCCCCTAACTCCGCCCAGTTCCGCCCATTCTCCGCCCCATGGCTGACTAA
TTTTTTTTATTTATGCAGAGGCCGAGGCCGCCTCTGCCTCTGAGCTATTCCAGAAGTAGTGAGGAGGCTTTTTTGGAGGCCTAGGCTTT
TGCAAAAAGCTCCCGGGAGCTTGTATATCCATTTTCGGATCTGATCAGCACGTGTTGACAATTAATCATCGGCATAGTATATCGGCATA
GTATAATACGACAAGGTGAGGAACTAAACCATGGCCAAGTTGACCAGTGCCGTTCCGGTGCTCACCGCGCGCGACGTCGCCGGAGCGGT
CGAGTTCTGGACCGACCGGCTCGGGTTCTCCCGGGACTTCGTGGAGGACGACTTCGCCGGTGTGGTCCGGGACGACGTGACCCTGTTCA
TCAGCGCGGTCCAGGACCAGGTGGTGCCGGACAACACCCTGGCCTGGGTGTGGGTGCGCGGCCTGGACGAGCTGTACGCCGAGTGGTCG
GAGGTCGTGTCCACGAACTTCCGGGACGCCTCCGGGCCGGCCATGACCGAGATCGGCGAGCAGCCGTGGGGGCGGGAGTTCGCCCTGCG
CGACCCGGCCGGCAACTGCGTGCACTTCGTGGCCGAGGAGCAGGACTGACACGTGCTACGAGATTTCGATTCCACCGCCGCCTTCTATG
AAAGGTTGGGCTTCGGAATCGTTTTCCGGGACGCCGGCTGGATGATCCTCCAGCGCGGGGATCTCATGCTGGAGTTCTTCGCCCACCCC
AACTTGTTTATTGCAGCTTATAATGGTTACAAATAAAGCAATAGCATCACAAATTTCACAAATAAAGCATTTTTTTCACTGCATTCTAG
TTGTGGTTTGTCCAAACTCATCAATGTATCTTATCATGTCTGTATACCGTCGACCTCTAGCTAGAGCTTGGCGTAATCATGGTCATAGC
TGTTTCCTGTGTGAAATTGTTATCCGCTCACAATTCCACACAACATACGAGCCGGAAGCATAAAGTGTAAAGCCTGGGGTGCCTAATGA
GTGAGCTAACTCACATTAATTGCGTTGCGCTCACTGCCCGCTTTCCAGTCGGGAAACCTGTCGTGCCAGCTGCATTAATGAATCGGCCA
ACGCGCGGGGAGAGGCGGTTTGCGTATTGGGCGCTCTTCCGCTTCCTCGCTCACTGACTCGCTGCGCTCGGTCGTTCGGCTGCGGCGAG
CGGTATCAGCTCACTCAAAGGCGGTAATACGGTTATCCACAGAATCAGGGGATAACGCAGGAAAGAACATGTGAGCAAAAGGCCAGCAA
AAGGCCAGGAACCGTAAAAAGGCCGCGTTGCTGGCGTTTTTCCATAGGCTCCGCCCCCCTGACGAGCATCACAAAAATCGACGCTCAAG
TCAGAGGTGGCGAAACCCGACAGGACTATAAAGATACCAGGCGTTTCCCCCTGGAAGCTCCCTCGTGCGCTCTCCTGTTCCGACCCTGC
CGCTTACCGGATACCTGTCCGCCTTTCTCCCTTCGGGAAGCGTGGCGCTTTCTCATAGCTCACGCTGTAGGTATCTCAGTTCGGTGTAG
GTCGTTCGCTCCAAGCTGGGCTGTGTGCACGAACCCCCCGTTCAGCCCGACCGCTGCGCCTTATCCGGTAACTATCGTCTTGAGTCCAA
CCCGGTAAGACACGACTTATCGCCACTGGCAGCAGCCACTGGTAACAGGATTAGCAGAGCGAGGTATGTAGGCGGTGCTACAGAGTTCT
TGAAGTGGTGGCCTAACTACGGCTACACTAGAAGAACAGTATTTGGTATCTGCGCTCTGCTGAAGCCAGTTACCTTCGGAAAAAGAGTT
GGTAGCTCTTGATCCGGCAAACAAACCACCGCTGGTAGCGGTGGTTTTTTTGTTTGCAAGCAGCAGATTACGCGCAGAAAAAAAGGATC
TCAAGAAGATCCTTTGATCTTTTCTACGGGGTCTGACGCTCAGTGGAACGAAAACTCACGTTAAGGGATTTTGGTCATGAGATTATCAA
AAAGGATCTTCACCTAGATCCTTTTAAATTAAAAATGAAGTTTTAAATCAATCTAAAGTATATATGAGTAAACTTGGTCTGACAGTTAC
CAATGCTTAATCAGTGAGGCACCTATCTCAGCGATCTGTCTATTTCGTTCATCCATAGTTGCCTGACTCCCCGTCGTGTAGATAACTAC
GATACGGGAGGGCTTACCATCTGGCCCCAGTGCTGCAATGATACCGCGAGACCCACGCTCACCGGCTCCAGATTTATCAGCAATAAACC
AGCCAGCCGGAAGGGCCGAGCGCAGAAGTGGTCCTGCAACTTTATCCGCCTCCATCCAGTCTATTAATTGTTGCCGGGAAGCTAGAGTA
AGTAGTTCGCCAGTTAATAGTTTGCGCAACGTTGTTGCCATTGCTACAGGCATCGTGGTGTCACGCTCGTCGTTTGGTATGGCTTCATT
CAGCTCCGGTTCCCAACGATCAAGGCGAGTTACATGATCCCCCATGTTGTGCAAAAAAGCGGTTAGCTCCTTCGGTCCTCCGATCGTTG
TCAGAAGTAAGTTGGCCGCAGTGTTATCACTCATGGTTATGGCAGCACTGCATAATTCTCTTACTGTCATGCCATCCGTAAGATGCTTT
TCTGTGACTGGTGAGTACTCAACCAAGTCATTCTGAGAATAGTGTATGCGGCGACCGAGTTGCTCTTGCCCGGCGTCAATACGGGATAA
TACCGCGCCACATAGCAGAACTTTAAAAGTGCTCATCATTGGAAAACGTTCTTCGGGGCGAAAACTCTCAAGGATCTTACCGCTGTTGA
GATCCAGTTCGATGTAACCCACTCGTGCACCCAACTGATCTTCAGCATCTTTTACTTTCACCAGCGTTTCTGGGTGAGCAAAAACAGGA
AGGCAAAATGCCGCAAAAAAGGGAATAAGGGCGACACGGAAATGTTGAATACTCATACTCTTCCTTTTTCAATATTATTGAAGCATTTA
TCAGGGTTATTGTCTCATGAGCGGATACATATTTGAATGTATTTAGAAAAATAAACAAATAGGGGTTCCGCGCACATTTCCCCGAAAAG
TGCCACCTGAC

Lentiviral plasmid DNA reconstruction: Whole plasmid sequence 4

(v) Frame vector

Vector name: Reverse-EF1a-eGFP-intron-frame / FUGW

Feature Name
CMV_immearly_promoter / HIV-1_5_LTR / truncHIV-1_3_LTR / HIV-1_psi_pack / RRE / cPPT / SV40 polyadenylation signal (Reverse) / WPRE (Reverse) / eGFP-intron (Reverse) 2 / mir-517a (-243 to +292) with cloning site (2x BsmBI) (Reverse) / eGFP-intron (Reverse) 1 / EF1a_promoter (Reverse) / U3PPT / HIV-1_5_LTR

GTCGACGGATCGGGAGATCTCCCGATCCCCTATGGTGCACTCTCAGTACAATCTGCTCTGATGCCGCATAGTTAAGCCAGTATCTGCTC
CCTGCTTGTGTGTTGGAGGTCGCTGAGTAGTGCGCGAGCAAAATTTAAGCTACAACAAGGCAAGGCTTGACCGACAATTGCATGAAGAA
TCTGCTTAGGGTTAGGCGTTTTGCGCTGCTTCGCGATGTACGGGCCAGATATACGCGTTGACATTGATTATTGACTAGTTATTAATAGT
AATCAATTACGGGGTCATTAGTTCATAGCCCATATATGGAGTTCCGCGTTACATAACTTACGGTAAATGGCCCGCCTGGCTGACCGCCC
AACGACCCCCGCCCATTGACGTCAATAATGACGTATGTTCCCATAGTAACGCCAATAGGGACTTTCCATTGACGTCAATGGGTGGAGTA
TTTACGGTAAACTGCCCACTTGGCAGTACATCAAGTGTATCATATGCCAAGTACGCCCCCTATTGACGTCAATGACGGTAAATGGCCCG
CCTGGCATTATGCCCAGTACATGACCTTATGGGACTTTCCTACTTGGCAGTACATCTACGTATTAGTCATCGCTATTACCATGGTGATG
CGGTTTTGGCAGTACATCAATGGGCGTGGATAGCGGTTTGACTCACGGGGATTTCCAAGTCTCCACCCCATTGACGTCAATGGGAGTTT
GTTTTGGCACCAAAATCAACGGGACTTTCCAAAATGTCGTAACAACTCCGCCCCATTGACGCAAATGGGCGGTAGGCGTGTACGGTGGG
AGGTC**TATATAA**GCAGCGCGTTTTGCCTGTACTGGGTCTCTCTGGTTAGACCAGATCTGAGCCTGGGAGCTCTCTGGCTAACTAGGGAA
CCCACTGCTTAAGCCTCAATAAAGCTTGCCTTGAGTGCTTCAAGTAGTGTGTGCCCGTCTGTTGTGTGACTCTGGTAACTAGAGATCCC
TCAGACCCTTTTAGTCAGTGTGGAAAATCTCTAGCAGTGGCGCCCGAACAGGGACTTGAAAGCGAAAGGGAAACCAGAGGAGCTCTCTC
GACGCAGGACTCGGCTTGCTGAAGCGCGCACGGCAAGAGGCGAGGGGCGGCGACTGGTGAGTACGCCAAAAATTTTGACTAGCGGAGGC
TAGAAGGAGAGAGATGGGTGCGAGAGCGTCAGTATTAAGCGGGGGAGAATTAGATCGCGATGGGAAAAAATTCGGTTAAGGCCAGGGGG
AAAGAAAAAATATAAATTAAAACATATAGTATGGGCAAGCAGGGAGCTAGAACGATTCGCAGTTAATCCTGGCCTGTTAGAAACATCAG
AAGGCTGTAGACAAATACTGGGACAGCTACAACCATCCCTTCAGACAGGATCAGAAGAACTTAGATCATTATATAATACAGTAGCAACC
CTCTATTGTGTGCATCAAAGGATAGAGATAAAAGACACCAAGGAAGCTTTAGACAAGATAGAGGAAGAGCAAAACAAAAGTAAGACCAC
CGCACAGCAAGCGGCCGCTGATCTTCAGACCTGGAGGAGGAGATATGAGGGACAATTGGAGAAGTGAATTATATAAATATAAAGTAGTA
AAAATTGAACCATTAGGAGTAGCACCCACCAAGGCAAAGAGAAGAGTGGTGCAGAGAGAAAAAAGAGCAGTGGGAATAGGAGCTTTGTT
CCTTGGGTTCTTGGGAGCAGCAGGAAGCACTATGGGCGCAGCGTCAATGACGCTGACGGTACAGGCCAGACAATTATTGTCTGGTATAG
TGCAGCAGCAGAACAATTTGCTGAGGGCTATTGAGGCGCAACAGCATCTGTTGCAACTCACAGTCTGGGGCATCAAGCAGCTCCAGGCA
AGAATCCTGGCTGTGGAAAGATACCTAAAGGATCAACAGCTCCTGGGGATTTGGGGTTGCTCTGGAAAACTCATTTGCACCACTGCTGT
GCCTTGGAATGCTAGTTGGAGTAATAAATCTCTGGAACAGATTTGGAATCACACGACCTGGATGGAGTGGGACAGAGAAATTAACAATT
ACACAAGCTTAATACACTCCTTAATTGAAGAATCGCAAAACCAGCAAGAAAAGAATGAACAAGAATTATTGGAATTAGATAAATGGGCA
AGTTTGTGGAATTGGTTTAACATAACAAATTGGCTGTGGTATATAAAATTATTCATAATGATAGTAGGAGGCTTGGTAGGTTTAAGAAT
AGTTTTTGCTGTACTTTCTATAGTGAATAGAGTTAGGCAGGGATATTCACCATTATCGTTTCAGACCCACCTCCCAACCCCGAGGGGAC
CCGACAGGCCCGAAGGAATAGAAGAAGAAGGTGGAGAGAGAGACAGAGACAGATCCATTCGATTAGTGAACGGATCGGCACTGCGTGCG
CCAATTCTGCAGACAAATGGCAGTATTCATCCACAATTTTAAAAGAAAAGGGGGGATTGGGGGGTACAGTGCAGGGGAAAGAATAGTAG
ACATAATAGCAACAGACATACAAACTAAAGAATTACAAAAACAAATTACAAAAATTCAAAATTTTCGGGTTTATTACAGGGACAGCAGA
GATCCAGTTTGGttaatacaaaccacaactagaatgcagtgaaaaaaatgctttattGGTACCTGAGGTGTGACTGGAAAACCCACCTC
CTCCTCCTCTTGTGCTTCTAGCCAGGCACAATCAGCATTGGTAGCTGCTGTATTGCTACTTGTGATTGCTCCATGTTTTTCTAGGTCTC
GAGGTCGACGGTATCGATGCGGGGAGGCGGCCCAAAGGGAGATCCGACTCGTCTGAGGGCGAAGGCGAAGACGCGGAAGAGGCCGCAGA
GCCGGCAGCAGGCCGCGGGAAGGAAGGTCCGCTGGATTGAGGGCCGAAGGGACGTAGCAGAAGGACGTCCCGCGCAGAATCCAGGTGGC
AACACAGGCGAGCAGCCAAGGAAAGGACGATGATTTCCCCGACAACACCACGGAATTGTCAGTGCCCAACAGCCGAGCCCCTGTCCAGC
AGCGGGCAAGGCAGGCGGCGATGAGTTCCGCCGTGGCAATAGGGAGGGGGAAAGCGAAAGTCCCGGAAAGGAGCTGACAGGTGGTGGCA
ATGCCCCAACCAGTGGGGGTTGCGTCAGCAAACACAGTGCACACCACGCCACGTTGCCTGACAACGGGCCACAACTCCTCATAAAGAGA
CAGCAACCAGGATTTATACAAGGAGGAGAAAATGAAAGCCATACGGGAAGCAATAGCATGATACAAAGGCATTAAAGCAGCGTATCCAC
ATAGCGTAAAAGGAGCAACATAGTTAAGAATACCAGTCAATCTTTCACAAATTTTGTAATCCAGAGGTTGATTATCGATAAGCTTGATA
TCGAATTCTTACTTGTACAGCTCGTCCATGCCGAGAGTGATCCCGGCGGCGGTCACGAACTCCAGCAGGACCATGTGATCGCGCTTCTC
GTTGGGGTCTTTGCTCAGGGCGGACTGGGTGCTCAGGTAGTGGTTGTCGGGCAGCAGCACGGGGCCGTCGCCGATGGGGGTGTTCTGCT
GGTAGTGGTCGGCGAGCTGCACGCTGCCGTCCTCGATGTTGTGGCGGATCTTGAAGTTCACCTTGATGCCGTTCTTCTGCTTGTCGGCC
ATGATATAGACGTTGTGGCTGTTGTAGTTGTACTCCAGCTTGTGCCCCAGGATGTTGCCGTCCTCCTTGAAGTCGATGCCCTTCAGCTC
GATGCGGTTCACCAGGGTGTCGCCCTCGAACTTCACCTGTGGAGAGAAAGGCAAAGTGGATGTCAGTAAGACCAATAGGTGCCTATCAT
GGCCGCGAGGTGGGAGAATCACTGGAACCCGAGAGGTGGAGTTTCCGGTGAGCAGAGATCCTGCTACTGCACTCCCAACGAAACAAGAG
TGTGTCTCAAAAAATCAAAAAATAAAAAGAGGACCACTTAAGCCCAAGAAATCTTGGTGGAAGGTGCTATCCACCCACGCCTGGGCAAT
AGAAAGCCTGTCTTGGAAAAATTTCAAATCACAAACAGAACCCCGACATCATGCAAGTCTGATGATAAGCACGCTCTGCAAATGTATTA
CCAAGATCAGCAGCATCTTCAACGTTGCTTGTCTCAAACAGTAGAGACGCATCTGTACGTCTCCTGCCTGAGATCTTCTTTTTTTTGAC
ACGGAGTCTCACTCTGCCGCCCAGGCTGGATGCGGTGGTGGGATCTCGGCTCATGGCAACTTCTGCCTCCTGGGTTCAAGCGATTCTTC
TGCGTCAGCCTCCTGAGTAGCTGGGATTAGAGGCACCCACCACCATGCATATTGCATATATGACTGCATATATGACCTGGTCATGCACC
CAAGATGTTGGTTTCATTTCCCCAGTGAAAACTCAAACCCCATGCACTCCTCGAAAACGCAAGAGTCTTCTCTGTCTCGACAAGCCCAG
TTTCTATTGGTCTCCTTAAACCTGTCTTGTAACCTTGATACTTACCTCGGCGCGGGTCTTGTAGTTGCCGTCGTCCTTGAAGAAGATGG
TGCGCTCCTGGACGTAGCCTTCGGGCATGGCGGACTTGAAGAAGTCGTGCTGCTTCATGTGGTCGGGGTAGCGGCTGAAGCACTGCACG
CCGTAGGTCAGGGTGGTCACGAGGGTGGGCCAGGGCACGGGCAGCTTGCCGGTGGTGCAGATGAACTTCAGGGTCAGCTTGCCGTAGGT

GGCATCGCCCTCGCCCTCGCCGGACACGCTGAACTTGTGGCCGTTTACGTCGCCGTCCAGCTCGACCAGGATGGGCACCACCCCGGTGA
ACAGCTCCTCGCCCTTGCTCACCATGGTGGCggatccCTCACGACACCTGAAATGGAAGAAAAAAACTTTGAACCACTGTCTGAGGCTT
GAGAATGAACCAAGATCCAAACTCAAAAAGGGCAAATTCCAAGGAGAATTACATCAAGTGCCAAGCTGGCCTAACTTCAGTCTCCACCC
ACTCAGTGTGGGGAAACTCCATCGCATAAAACCCCTCCCCCCAACCTAAAGACGACGTACTCCAAAAGCTCGAGAACTAATCGAGGTGC
CTGGACGGCGCCCGGTACTCCGTGGAGTCACATGAAGCGACGGCTGAGGACGGAAAGGCCCTTTTCCTTTGTGTGGGTGACTCACCCGC
CCGCTCTCCCGAGCGCCGCGTCCTCCATTTTGAGCTCCCTGCAGCAGGGCCGGGAAGCGGCCATCTTTCCGCTCACGCAACTGGTGCCG
ACCGGGCCAGCCTTGCCGCCCAGGGCGGGGCGATACACGGCGGCGCGAGGCCAGGCACCAGAGCAGGCCGGCCAGCTTGAGACTACCCC
CGTCCGATTCTCGGTGGCCGCGCTCGCAGGCCCCGCCTCGCCGAACATGTGCGCTGGGACGCACGGGCCCCGTCGCCGCCCGCGGCCCC
AAAAACCGAAATACCAGTGTGCAGATCTTGGCCCGCATTTACAAGACTATCTTGCCAGAAAAAAAGCGTCGCAGCAGGTCATCAAAAAT
TTTAAATGGCTAGAGACTTATCGAAAGCAGCGAGACAGGCGCGAAGGTGCCACCAGATTCGCACGCGGCGGCCCCAGCGCCCAGGCCAG
GCCTCAACTCAAGCACGAGGCGAAGGGGCTCCTTAAGCGCAAGGCCTCGAACTCTCCCACCCACTTCCAACCCGAAGCTCGGGATCAAG
AATCACGTACTGCAGCCAGGGGCGTGGAAGTAATTCAAGGCACGCAAGGGCCATAACCCGTAAAGAGGCCAGGCCCGCGGGAACCACAC
ACGGCACTTACCTGTGTTCTGGCGGCAAACCCGTTGCGAAAAAGAACGTTCACGGCGACTACTGCACTTATATACGGTTCTCCCCCACC
CTCGGGAAAAAGGCGGAGCCAGTACACGACATCACTTTCCCAGTTTACCCCGCGCCACCTTCTCTAGGCACCGGTTCAATTGCCGACCC
CTCCCCCCAACTTCTCGGGGACTGTGGGCGATGTGCGCTCTGCCCACTGACGGGCACCGGAGCCTCACGTTAATTAAGTACCTTTAAGA
CCAATGACTTACAAGGCAGCTGTAGATCTTAGCCACTTTTTAAAAGAAAAGGGGGGACTGGAAGGGCTAATTCACTCCCAACGAAGACA
AGATATCCTTGATCTGTGGATCTACCACACACAAGGCTACTTCCCTGATTGGCAGAACTACACACCAGGGCCAGGGATCAGATATCCAC
TGACCTTTGGATGGTGCTACAAGCTAGTACCAGTTGAGCAAGAGAAGGTAGAAGAAGCCAATGAAGGAGAGAACACCCGCTTGTTACAC
CCTGTGAGCCTGCATGGGATGGATGACCCGGAGAGAGAAGTATTAGAGTGGAGGTTTGACAGCCGCCTAGCATTTCATCACATGGCCCG
AGAGCTGCATCCGGACTGTACTGGGTCTCTCTGGTTAGACCAGATCTGAGCCTGGGAGCTCTCTGGCTAACTAGGGAACCCACTGCTTA
AGCCTCAATAAAGCTTGCCTTGAGTGCTTCAAGTAGTGTGTGCCCGTCTGTTGTGTGACTCTGGTAACTAGAGATCCCTCAGACCCTTT
TAGTCAGTGTGGAAAATCTCTAGCAGGGCCCGTTTAAACCCGCTGATCAGCCTCGACTGTGCCTTCTAGTTGCCAGCCATCTGTTGTTT
GCCCCTCCCCCGTGCCTTCCTTGACCCTGGAAGGTGCCACTCCCACTGTCCTTTCCTAATAAAATGAGGAAATTGCATCGCATTGTCTG
AGTAGGTGTCATTCTATTCTGGGGGGTGGGGTGGGGCAGGACAGCAAGGGGGAGGATTGGGAAGACAATAGCAGGCATGCTGGGGATGC
GGTGGGCTCTATGGCTTCTGAGGCGGAAAGAACCAGCTGGGGCTCTAGGGGGTATCCCCACGCGCCCTGTAGCGGCGCATTAAGCGCGG
CGGGTGTGGTGGTTACGCGCAGCGTGACCGCTACACTTGCCAGCGCCCTAGCGCCCGCTCCTTTCGCTTTCTTCCCTTCCTTTCTCGCC
ACGTTCGCCGGCTTTCCCCGTCAAGCTCTAAATCGGGGGCTCCCTTTAGGGTTCCGATTTAGTGCTTTACGGCACCTCGACCCCAAAAA
ACTTGATTAGGGTGATGGTTCACGTAGTGGGCCATCGCCCTGATAGACGGTTTTTCGCCCTTTGACGTTGGAGTCCACGTTCTTTAATA
GTGGACTCTTGTTCCAAACTGGAACAACACTCAACCCTATCTCGGTCTATTCTTTTGATTTATAAGGGATTTTGCCGATTTCGGCCTAT
TGGTTAAAAAATGAGCTGATTTAACAAAAATTTAACGCGAATTAATTCTGTGGAATGTGTGTCAGTTAGGGTGTGGAAAGTCCCCAGGC
TCCCCAGCAGGCAGAAGTATGCAAAGCATGCATCTCAATTAGTCAGCAACCAGGTGTGGAAAGTCCCCAGGCTCCCCAGCAGGCAGAAG
TATGCAAAGCATGCATCTCAATTAGTCAGCAACCATAGTCCCGCCCCTAACTCCGCCCATCCCGCCCCTAACTCCGCCCAGTTCCGCCC
ATTCTCCGCCCCATGGCTGACTAATTTTTTTTATTTATGCAGAGGCCGAGGCCGCCTCTGCCTCTGAGCTATTCCAGAAGTAGTGAGGA
GGCTTTTTTGGAGGCCTAGGCTTTTGCAAAAAGCTCCCGGGAGCTTGTATATCCATTTTCGGATCTGATCAGCACGTGTTGACAATTAA
TCATCGGCATAGTATATCGGCATAGTATAATACGACAAGGTGAGGAACTAAACCATGGCCAAGTTGACCAGTGCCGTTCCGGTGCTCAC
CGCGCGCGACGTCGCCGGAGCGGTCGAGTTCTGGACCGACCGGCTCGGGTTCTCCCGGGACTTCGTGGAGGACGACTTCGCCGGTGTGG
TCCGGGACGACGTGACCCTGTTCATCAGCGCGGTCCAGGACCAGGTGGTGCCGGACAACACCCTGGCCTGGGTGTGGGTGCGCGGCCTG
GACGAGCTGTACGCCGAGTGGTCGGAGGTCGTGTCCACGAACTTCCGGGACGCCTCCGGGCCGGCCATGACCGAGATCGGCGAGCAGCC
GTGGGGGCGGGAGTTCGCCCTGCGCGACCCGGCCGGCAACTGCGTGCACTTCGTGGCCGAGGAGCAGGACTGACACGTGCTACGAGATT
TCGATTCCACCGCCGCCTTCTATGAAAGGTTGGGCTTCGGAATCGTTTTCCGGGACGCCGGCTGGATGATCCTCCAGCGCGGGGATCTC
ATGCTGGAGTTCTTCGCCCACCCCAACTTGTTTATTGCAGCTTATAATGGTTACAAATAAAGCAATAGCATCACAAATTTCACAAATAA
AGCATTTTTTTCACTGCATTCTAGTTGTGGTTTGTCCAAACTCATCAATGTATCTTATCATGTCTGTATACCGTCGACCTCTAGCTAGA
GCTTGGCGTAATCATGGTCATAGCTGTTTCCTGTGTGAAATTGTTATCCGCTCACAATTCCACACAACATACGAGCCGGAAGCATAAAG
TGTAAAGCCTGGGGTGCCTAATGAGTGAGCTAACTCACATTAATTGCGTTGCGCTCACTGCCCGCTTTCCAGTCGGGAAACCTGTCGTG
CCAGCTGCATTAATGAATCGGCCAACGCGCGGGGAGAGGCGGTTTGCGTATTGGGCGCTCTTCCGCTTCCTCGCTCACTGACTCGCTGC
GCTCGGTCGTTCGGCTGCGGCGAGCGGTATCAGCTCACTCAAAGGCGGTAATACGGTTATCCACAGAATCAGGGGATAACGCAGGAAAG
AACATGTGAGCAAAAGGCCAGCAAAAGGCCAGGAACCGTAAAAAGGCCGCGTTGCTGGCGTTTTTCCATAGGCTCCGCCCCCCTGACGA
GCATCACAAAAATCGACGCTCAAGTCAGAGGTGGCGAAACCCGACAGGACTATAAAGATACCAGGCGTTTCCCCCTGGAAGCTCCCTCG
TGCGCTCTCCTGTTCCGACCCTGCCGCTTACCGGATACCTGTCCGCCTTTCTCCCTTCGGGAAGCGTGGCGCTTTCTCATAGCTCACGC
TGTAGGTATCTCAGTTCGGTGTAGGTCGTTCGCTCCAAGCTGGGCTGTGTGCACGAACCCCCCGTTCAGCCCGACCGCTGCGCCTTATC
CGGTAACTATCGTCTTGAGTCCAACCCGGTAAGACACGACTTATCGCCACTGGCAGCAGCCACTGGTAACAGGATTAGCAGAGCGAGGT
ATGTAGGCGGTGCTACAGAGTTCTTGAAGTGGTGGCCTAACTACGGCTACACTAGAAGAACAGTATTTGGTATCTGCGCTCTGCTGAAG
CCAGTTACCTTCGGAAAAAGAGTTGGTAGCTCTTGATCCGGCAAACAAACCACCGCTGGTAGCGGTGGTTTTTTTGTTTGCAAGCAGCA
GATTACGCGCAGAAAAAAAGGATCTCAAGAAGATCCTTTGATCTTTTCTACGGGGTCTGACGCTCAGTGGAACGAAAACTCACGTTAAG
GGATTTTGGTCATGAGATTATCAAAAAGGATCTTCACCTAGATCCTTTTAAATTAAAAATGAAGTTTTAAATCAATCTAAAGTATATAT
GAGTAAACTTGGTCTGACAGTTACCAATGCTTAATCAGTGAGGCACCTATCTCAGCGATCTGTCTATTTCGTTCATCCATAGTTGCCTG
ACTCCCCGTCGTGTAGATAACTACGATACGGGAGGGCTTACCATCTGGCCCCAGTGCTGCAATGATACCGCGAGACCCACGCTCACCGG
CTCCAGATTTATCAGCAATAAACCAGCCAGCCGGAAGGGCCGAGCGCAGAAGTGGTCCTGCAACTTTATCCGCCTCCATCCAGTCTATT
AATTGTTGCCGGGAAGCTAGAGTAAGTAGTTCGCCAGTTAATAGTTTGCGCAACGTTGTTGCCATTGCTACAGGCATCGTGGTGTCACG
CTCGTCGTTTGGTATGGCTTCATTCAGCTCCGGTTCCCAACGATCAAGGCGAGTTACATGATCCCCCATGTTGTGCAAAAAAGCGGTTA
GCTCCTTCGGTCCTCCGATCGTTGTCAGAAGTAAGTTGGCCGCAGTGTTATCACTCATGGTTATGGCAGCACTGCATAATTCTCTTACT
GTCATGCCATCCGTAAGATGCTTTTCTGTGACTGGTGAGTACTCAACCAAGTCATTCTGAGAATAGTGTATGCGGCGACCGAGTTGCTC
TTGCCCGGCGTCAATACGGGATAATACCGCGCCACATAGCAGAACTTTAAAAGTGCTCATCATTGGAAAACGTTCTTCGGGGCGAAAAC
TCTCAAGGATCTTACCGCTGTTGAGATCCAGTTCGATGTAACCCACTCGTGCACCCAACTGATCTTCAGCATCTTTTACTTTCACCAGC

GTTTCTGGGTGAGCAAAAACAGGAAGGCAAAATGCCGCAAAAAAGGGAATAAGGGCGACACGGAAATGTTGAATACTCATACTCTTCCT
TTTTCAATATTATTGAAGCATTTATCAGGGTTATTGTCTCATGAGCGGATACATATTTGAATGTATTTAGAAAAATAAACAAATAGGGG
TTCCGCGCACATTTCCCCGAAAAGTGCCACCTGAC

Supplementary Table S1: Lentiviral plasmids for DNA reconstruction

**(i) Promoter for the expression cassette**			
EF1a forward	(PacI)-(EF1a forward)	CCttaattaaCGTGAGGCTCCGGTGC	
EF1a reverse	(BamHI)-(EF1a reverse)	CGCggatccCTCACGACACCTGAAATGGAAG	
**(ii) eGFP-intron splicing system**			
EGFP forward from start codon	(BamHI)-(Kozak)-(EGFP forward from start codon)	CGCggatccGCCACCATGGTGAGCAAGGGCGA	
intron-eGFP antisense	Refer Biotechnol Lett (2011) 33:1723-1728	GTGTCGCCCTCGAACTTCACCTGTGGAGAGAAAGGC A	
intron-eGFP sense	Refer Biotechnol Lett (2011) 33:1723-1728	ACTACAAGACCCGCGCCGAGGTAAGTATCAAGGTTA C	
EGFP reverse from stop codon	(EcoRI)-(EGFP reverse from stop codon)	CCGgaattcTTACTTGTACAGCTCGTCCATGC	
Chemically-synthesized intron forward	Refer Biotechnol Lett (2011) 33:1723-1728 / Two BsmBI sites	GTAAGTATCAAGGTTACAAGACAGGTTTAAGGAGAC CAATAGAAACTGGGCTTGTCGAGACAGAGAAGACTC TTGCGTTTTCGATGAGAC	
Chemically-synthesized intron reverse	Refer Biotechnol Lett (2011) 33:1723-1728 / Two BsmBI sites	CTGTGGAGAGAAAGGCAAAGTGGATGTCAGTAAGA CCAATAGGTGCCTATCATGGCCTGAGACGCATCTGTA CGTCTCATCGAAAACGCA	
**(iii)-1 pre-miRNA vector**			
pre-mir-517a forward	(XhoI)-(pre-mir-517a forward)	ccgCTCGAGagactccgtgtcaaaaaaaagaagaTCTCAGGC AGTGACCCTCTAGATGGAAGCACTGTCTGTTGTATAA AAGAAAAGATCGTGC	PCR without template
pre-mir-517a reverse	(NotI)-(pre-mir-517a reverse)	gtaGCGGCCGCtcagcagcatcttcaacgttgcttgTCTCAAA CAGTAACACTCTAAAGGGATGCACGATCTTTTCTTTT ATACAACAG	PCR without template
pre-mir-525 forward	(XhoI)-(pre-mir-525 forward)	ccgCTCGAGacccacggtgctggagcaagaagatCTCAAGCT GTGACTCTCCAGAGGGATGCACTTTCTCTTATGTGAA AAAAAAGAA	PCR without template
pre-mir-525 reverse	(NotI)-(pre-mir-525 reverse)	gtaGCGGCCGCcagcatcaacttcaacgttgctttaCCCAAAC CGTAACGCTCTAAAGGGAAGCGCCTTCTTTTTTTTCA CATAAGAG	PCR without template
pre-mir-14 forward	(XhoI)-(pre-mir-14 forward)	ccgCTCGAGcaacaacgtaacgtactgcaacctaTGTGGGAG CGAGACGGGGACTCACTGTGCTTATTAAATAGTCAG TC	PCR without template
pre-mir-14 reverse	(NotI)-(pre-mir-14 reverse)	gtaGCGGCCGCgccatgatgatacgcccgcaatttgTATAGGA GAGAGAAAAAGACTGACTATTTAATAAGCACAGTGA G	PCR without template
pre-mir-276a forward	(XhoI)-(pre-mir-276a forward)	ccgCTCGAGaaacgcgctgccaagatattttttaCCTGGTTTTT GCCATCAGCGAGGTATAGAGTTCCTACGTTCATTATA AACTCGTAGGAAC	PCR without template
pre-mir-276a reverse	(NotI)-(pre-mir-276a reverse)	gtaGCGGCCGCatactttttgggatttttgcattcaCTTGGTTG TTTTTTGGTCTTCCAAGAGCACGGTATGAAGTTCCTA CGAGTTTATAATGAACG	PCR without template
pre-mir-77 forward	(XhoI)-(pre-mir-77 forward)	ccgCTCGAGtctatattctggtaagtttttttttGCATCTGCCAA ACCGCCCGTTTGGATGGTTGTGCTCTGAGGAAATAC GCACAGAATGTCATTTC	PCR without template
pre-mir-77 reverse	(NotI)-(pre-mir-77 reverse)	gtaGCGGCCGCaactccaataactgattcaacattcCAAACTC TATACCAATTTGGACAGCTATGGCCTGATGAAATGAC ATTCTGTGCGTATTTC	PCR without template

pre-mir-230 forward	(XhoI)-(pre-mir-230 forward)	ccgCTCGAGtctgcttgtatttttttgtctgatgTTGATGTAATGCCGTCACTTGGTC	PCR with genome template
pre-mir-230 reverse	(NotI)-(pre-mir-230 reverse)	gtaGCGGCCGCcgattatgaaaatgaatatttaagaAATATGCGAATACCGTCTCCTGG	PCR with genome template
pre-mir-107 forward	(XhoI)-(pre-mir-107 forward)	aaccgCTCGAGcttccacccgcaatactcg	PCR with genome template
pre-mir-107 reverse	(NotI)-(pre-mir-107 reverse)	gtaGCGGCCGCctctggctggccttggct	PCR with genome template
pre-mir-122 forward	(XhoI)-(pre-mir-122 forward)	ccgCTCGAGgtgcctgacagactttccttagcagAGCTGTGGAGTGTGACAATGGTGTTTGTGTCCAAACCATCAAAC	PCR without template
pre-mir-122 reverse	(NotI)-(pre-mir-122 reverse)	gtaGCGGCCGCtggagtggacggattgcctagcagtAGCTATTTAGTGTGATAATGGCGTTTGATGGTTTGGACACAAAC	PCR without template
(iii)-2 pri-miRNA vector			
pri-mir-517a forward	(XhoI)-(pri-mir-517a forward)	aaccgCTCGAGgagtgcatggggtttgagtt	
pri-mir-517a reverse	(NotI)-(pri-mir-517a reverse)	gtaGCGGCCGCgaggtgggagaatcactgga	
pri-mir-525 forward	(XhoI)-(pri-mir-525 forward)	aaccgCTCGAGttttctttctggaggcgaaa	
pri-mir-525 reverse	(NotI)-(pri-mir-525 reverse)	gtaGCGGCCGCctcacgcctgtaatcccaat	
pri-mir-2b-1 forward	(XhoI)-(pri-mir-2b-1 forward)	ccgCTCGAGgcaaaatccttgcagtagcc	
pri-mir-2b-1 reverse	(NotI)-(pri-mir-2b-1 reverse)	gtaGCGGCCGCggccattaagcaatataaacca	
pri-mir-2b-2 forward	(XhoI)-(pri-mir-2b-2 forward)	ccgCTCGAGttgcaatcaacatttgcgta	
pri-mir-2b-2 reverse	(NotI)-(pri-mir-2b-2 reverse)	gtaGCGGCCGCtgactgcgggagttcagaat	
pri-mir-14 forward	(XhoI)-(pri-mir-14 forward)	ccgCTCGAGgttcgttcagcttggctttc	
pri-mir-14 reverse	(NotI)-(pri-mir-14 reverse)	gtaGCGGCCGCtgcgtttgattattcgtgga	
pri-mir-276a forward	(XhoI)-(pri-mir-276a forward)	ccgCTCGAGctagaaattcccccagcaca #PCR product has XhoI site at 229/233, Cloning worked, though.	
pri-mir-276a reverse	(NotI)-(pri-mir-276a reverse)	ataagaatGCGGCCGCttgggcaaacacactactgaa #PCR product has XhoI site at 229/233, Cloning worked though.	
pri-mir-10b forward	(XhoI)-(pri-mir-10b forward)	ccgCTCGAGggtggctgtgctgaagagat	
pri-mir-10b reverse	(NotI)-(pri-mir-10b reverse)	gtaGCGGCCGCttctggcctttcacctcact	
pri-mir-107 forward	(XhoI)-(pri-mir-107 forward)	ccgCTCGAGaagggtacagcgagtgagga	
pri-mir-107 reverse	(NotI)-(pri-mir-107 reverse)	gtaGCGGCCGCttcactcgccaagctctttt	
pri-mir-122 forward	(XhoI)-(pri-mir-122 forward)	ccgCTCGAGggaaaccatgacgaggtgag	
pri-mir-122 reverse	(NotI)-(pri-mir-122 reverse)	gtaGCGGCCGCgagggtcccataggagaggt	
**(iv) forward vector / reverse vector**			
MCS for reverse vector in FUGW expression cassette (top)	(SV40 pA Rev)-(KpnI)-(XbaI)-(EcoRI)-(NheI)-(BamHI)-(SbfI)-(PacI)-(KpnI End)	acaaaccacaactagaatgcagtgaaaaaaatgctttattggtaccTCTAGAgaattcGCTAGCggatccCCTGCAGGttaattaaGTAC	
MCS for reverse vector in FUGW expression cassette (bottom)	(PacI)-(SbfI)-(BamHI)-(NheI)-(EcoRI)-(XbaI)-(KpnI)-(SV40 pA)-(PacI end)	ttaattaaCCTGCAGGggatccGCTAGCgaattcTCTAGAggtaccaataaagcatttttttcactgcattctagttgtggtttgtAT	
**(v) frame vector**			
mir-517a frame forward-1	(XhoI)-(mir-517a forward-1)	AACCGCTCGAGGAGTGCATGGGGTTTGAGTT	
mir-517a frame reverse-1	(BsmBI)-(BsmBI)-(mir-517a reverse-1)	GAGACGCATCTGTACGTCTCCTGCCTGAGATCTTCTTTTTTTTG	
mir-517a frame forward-2	(BsmBI)-(BsmBI)-(mir-517a forward-2)	GAGACGTACAGATGCGTCTCTACTGTTTGAGACAAGCAACGTTG	
mir-517a frame reverse-2	(NotI)-(mir-517a reverse-2)	GTAGCGGCCGCGAGGTGGGAGAATCACTGGA	
dme-pre-mir-2b-1 in frame (top)	(5' ligation site)-(5' arm)-(mir-517a loop)-(3' arm)-(3' ligation site)	ggcagtgTGTCTTCAAAGTGGCAGTGACATGgttgtataaaagaaaTATTCATATCACAGCCAGCTTTGAGGAGCt	

dme-pre-mir-2b-1 in frame (bottom)	(3' ligation site)-(3' arm)-(mir-517a loop)-(5' arm)-(5' ligation site)	cagtaGCTCCTCAAAGCTGGCTGTGATATGAATAtttctt ttatacaacCATGTCACTGCCACTTTGAAGACAcac
dme-pre-mir-14 in frame (top)	(5' ligation site)-(5' arm)-(mir-517a loop)-(3' arm)-(3' ligation site)	ggcagtgGTGGGAGCGAGACGGGGACTCACTgttgtata aaagaaaAGTCAGTCTTTTTCTCTCTCCTATt
dme-pre-mir-14 in frame (bottom)	(3' ligation site)-(3' arm)-(mir-517a loop)-(5' arm)-(5' ligation site)	cagtaATAGGAGAGAGAAAAAGACTGACTtttcttttatac aacAGTGAGTCCCCGTCTCGCTCCCACcac
dme-pre-mir-276a in frame (top)	(5' ligation site)-(5' arm)-(mir-517a loop)-(3' arm)-(3' ligation site)	ggcagtgTCAGCGAGGTATAGAGTTCCTACGgttgtataa aagaaaCGTAGGAACTTCATACCGTGCTCTt
dme-pre-mir-276a in frame (bottom)	(3' ligation site)-(3' arm)-(mir-517a loop)-(5' arm)-(5' ligation site)	cagtaAGAGCACGGTATGAAGTTCCTACGtttcttttatac aacCGTAGGAACTCTATACCTCGCTGAcac
mmu-pre-mir-10b in frame (top)	(5' ligation site)-(5' arm)-(mir-517a loop)-(3' arm)-(3' ligation site)	ggcagtgTATACCCTGTAGAACCGAATTTGTGgttgtataa aagaaaCACAGATTCGATTCTAGGGGAATAt
mmu-pre-mir-10b in frame (bottom)	(3' ligation site)-(3' arm)-(mir-517a loop)-(5' arm)-(5' ligation site)	cagtaTATTCCCCTAGAATCGAATCTGTGtttcttttataca acCACAAATTCGGTTCTACAGGGTATAcac
mmu-pre-mir-122 in frame (top)	(5' ligation site)-(5' arm)-(mir-517a loop)-(3' arm)-(3' ligation site)	ggcagtgTGGAGTGTGACAATGGTGTTTGgttgtataaaa gaaaCAAACGCCATTATCACACTAAAt
mmu-pre-mir-122 in frame (bottom)	(3' ligation site)-(3' arm)-(mir-517a loop)-(5' arm)-(5' ligation site)	cagtaTTTAGTGTGATAATGGCGTTTGtttcttttatacaac CAAACACCATTGTCACACTCCAcac
mmu-pre-mir-107 in frame (top)	(5' ligation site)-(5' arm)-(mir-517a loop)-(3' arm)-(3' ligation site)	ggcagtgTCAGCTTCTTTACAGTGTTGCCTTGgttgtataa aagaaaCAAGCAGCATTGTACAGGGCTATCAt
mmu-pre-mir-107 in frame (bottom)	(3' ligation site)-(3' arm)-(mir-517a loop)-(5' arm)-(5' ligation site)	cagtaTGATAGCCCTGTACAATGCTGCTTGtttcttttatac aacCAAGGCAACACTGTAAAGAAGCTGAcac
mmu-pre-mir-187 in frame (top)	(5' ligation site)-(5' arm)-(mir-517a loop)-(3' arm)-(3' ligation site)	ggcagtgCAGGCTACAACACAGGACCCGGGgttgtataaa agaaaCCTCGTGTCTTGTGTTGCAGCCGGt
mmu-pre-mir-187 in frame (bottom)	(3' ligation site)-(3' arm)-(mir-517a loop)-(5' arm)-(5' ligation site)	cagtaCCGGCTGCAACACAAGACACGAGGtttcttttatac aacCCCGGGTCCTGTGTTGTAGCCTGcac
mmu-pre-mir-675 in frame (top)	(5' ligation site)-(5' arm)-(mir-517a loop)-(3' arm)-(3' ligation site)	ggcagtgACTGGTGCGGAAAGGGCCCACAGTgttgtata aaagaaaACTGTATGCCCTAACCGCTCAGTt
mmu-pre-mir-675 in frame (bottom)	(3' ligation site)-(3' arm)-(mir-517a loop)-(5' arm)-(5' ligation site)	cagtaACTGAGCGGTTAGGGCATACAGTtttcttttataca acACTGTGGGCCCTTTCCGCACCAGTcac
mmu-pre-mir-1192 in frame (top)	(5' ligation site)-(5' arm)-(mir-517a loop)-(3' arm)-(3' ligation site)	ggcagtgAAACAAACAAACAGACCAAATTgttgtataaaa gaaaGATTTGCTCTTTTTGTTTGTTTt
mmu-pre-mir-1192 in frame (bottom)	(3' ligation site)-(3' arm)-(mir-517a loop)-(5' arm)-(5' ligation site)	cagtaAAACAAACAAAAAGAGCAAATCtttcttttatacaac AATTTGGTCTGTTTGTTTGTTTcac
cel-pre-mir-77 in frame (top)	(5' ligation site)-(5' arm)-(mir-517a loop)-(3' arm)-(3' ligation site)	ggcagtgTGGATGGTTGTGCTCTGAGGAAATgttgtataa aagaaaATTTCATCAGGCCATAGCTGTCCAt
cel-pre-mir-77 in frame (bottom)	(3' ligation site)-(3' arm)-(mir-517a loop)-(5' arm)-(5' ligation site)	cagtaTGGACAGCTATGGCCTGATGAAATtttcttttatac aacATTTCCTCAGAGCACAACCATCCAcac
cel-pre-mir-230 in frame (top)	(5' ligation site)-(5' arm)-(mir-517a loop)-(3' arm)-(3' ligation site)	ggcagtgTCACTTGGTCGGCGATTTAATATTAgttgtataa aagaaaTAGTATTAGTTGTGCGACCAGGAGAt
cel-pre-mir-230 in frame (bottom)	(3' ligation site)-(3' arm)-(mir-517a loop)-(5' arm)-(5' ligation site)	cagtaTCTCCTGGTCGCACAACTAATACTAtttcttttatac aacTAATATTAAATCGCCGACCAAGTGAcac
**(vi)-1 truncated pri-miRNA vector (mir-517a)**		
mir-517a forward (-25 to -1)	(XhoI)-(mir-517a forward) From -25	aaccgCTCGAGagactccgtgtcaaaaaaaagaaga
mir-517a forward (-30 to -8)	(XhoI)-(mir-517a forward) From -30	aaccgCTCGAGgagtgagactccgtgtcaaaaaa
mir-517a forward (-62 to -45)	(XhoI)-(mir-517a forward) From -62	aaccgCTCGAGagatcccaccaccgcatc
mir-517a forward (-93 to -74)	(XhoI)-(mir-517a forward) From -93	aaccgCTCGAGgaacccaggaggcagaagtt
mir-517a forward (-243 to -219) = pri-mir-517a forward	(XhoI)-(mir-517a forward) From -243	aaccgCTCGAGgagtgcatggggtttgagtt
mir-517a reverse (+25 to +5)	(NotI)-(mir-517a reverse) To +25	gtaGCGGCCGCtcagcagcatcttcaacgttgcttg

mir-517a reverse (+53 to +36)	(NotI)-(mir-517a reverse) To +53	gtaGCGGCCGCagcacgctctgcaaatgt
mir-517a reverse (+90 to +71)	(NotI)-(mir-517a reverse) To +90	gtaGCGGCCGCacaaacagaaccccgacatc
mir-517a reverse (+292 to +273) = pri-mir-517a reverse	(NotI)-(pri-mir-517a reverse) From +292	gtaGCGGCCGCgaggtgggagaatcactgga
**(vi)-2 truncated pri-miRNA vector (mir-525)**		
mir-525 forward (-25 to -7)	(XhoI)-(mir-525 forward) From -25	aaccgCTCGAGacccacggtgctggagcaa
mir-525 forward (-30 to -9)	(XhoI)-(mir-525 forward) From -30	aaccgCTCGAGcaaaaacccacggtgctg
mir-525 forward (-102 to -83)	(XhoI)-(mir-525 forward) From -102	aaccgCTCGAGacatggaaacagggccaata
mir-525 forward (-225 to -206) = pri-mir-525 forward	(XhoI)-(mir-525 forward) From -225	aaccgCTCGAGttttctttctggaggcgaaa
mir-525 reverse (+25 to +5)	(NotI)-(mir-525 forward) To +25	gtaGCGGCCGCcagcatcaacttcaacgttgc
mir-525 reverse (+85 to +66)	(NotI)-(mir-525 forward) To +85	gtaGCGGCCGCgaaacccaccatcatccaag
**(vii) mir-517a (-25 to +292) without Pabp binding site (AAAAA(-))**		
mir-517a (-25 to -1) AAAAA(-) forward	(XhoI)-(-25 to -14)-(In:TGGAGC, Out:AAAAAA)-(mir-517a forward) From -7	AACCGctcgagAGACTCCGTGTCtggagcAAGAAGATCT CAGGCAGTGACC
mir-517a reverse (+292 to +273) = pri-mir-517a reverse	(NotI)-(pri-mir-517a reverse) From +292	gtaGCGGCCGCgaggtgggagaatcactgga
mir-517a forward (-25 to -1)	(XhoI)-(mir-517a forward) From -25	aaccgCTCGAGagactccgtgtcaaaaaaaagaaga
mir-517a reverse (+292 to +273) = pri-mir-517a reverse	(NotI)-(pri-mir-517a reverse) From +292	gtaGCGGCCGCgaggtgggagaatcactgga
**(viii) Pabp family expression vector**		
MCS (top)	(BamHI end)-(SbfI)-(AscI)-(XbaI)-(BstBI)-(NheI)-(AsiSI)-(EcoRI end)	GATCCCCTGCAGGGGCGCGCCTCTAGATTCGAAGCT AGCGGCGCGCCG
MCS (bottom)	(EcoRI end)-(AsiSI)-(NheI)-(BstBI)-(XbaI)-(AscI)-(SbfI)-(BamHI end)	AATTCGGCGCGCCGCTAGCTTCGAATCTAGAGGCGC GCCCCTGCAGGG
mCherry forward from start codon	(BamHI)-(Kozak)-(EGFP/mCherry/EBFP2 start)	CGCggatccGCCACCATGGTGAGCAAGGGCGA
mCherry reverse from stop codon	(EcoRI)-(EGFP/mCherry/EBFP2 reverse from stop codon)	CCGgaattcTTACTTGTACAGCTCGTCCATGC
mCherry forward from start codon	(NheI)-(EGFP/mCherry/EBFP2 forward from ATG)	AACATGgctagcATGGTGAGCAAGGGCGA
mCherry reverse from stop codon	(EcoRI)-(EGFP/mCherry/EBFP2 reverse from stop codon)	CCGgaattcTTACTTGTACAGCTCGTCCATGC
Flexible linker in fusion protein (top)	(AscI End)-(XbaI)-(2x GGGGS)-(NheI End)	CGCGCCtctagaGGCGGAGGTGGCAGCGGCGGAGGT GGCAGCg
Flexible linker in fusion protein (bottom)	(NheI End)-(2x GGGGS)-(XbaI)-(AscI End)	ctagcGCTGCCACCTCCGCCGCTGCCACCTCCGCCtcta gaGG
mPabpn1 CDS forward	(BamHI)-(Kozak)-(Flag)-(forward primer from start codon)	cgcGGATCCgccaccatggattacaaggatgacgacgataagAT GGCGGCGGCGGCGGCG
mPabpn1 CDS reverse	(XbaI)-(reverse primer from the last codon before stop codon)	ACGTtctagaGTAAGGGGAATACCATGATGTCG
mPabpc1 CDS forward	(BamHI)-(Kozak)-(Flag)-(forward primer from start codon)	cgcGGATCCgccaccatggattacaaggatgacgacgataagAT GAACCCCAGCGCCCCC
mPabpc1 CDS reverse	(XbaI)-(reverse primer from the last codon before stop codon)	ACGTtctagaGACAGTTGGAACACCAGTGGC
mPabpc4 CDS forward	(BglII)-(Kozak)-(Flag)-(forward primer from start codon)	ggaAGATCTgccaccatggattacaaggatgacgacgataagAT GAACGCTGCAGCCAGC

mPabpc4 CDS reverse	(XbaI)-(reverse primer from the last codon before stop codon)	ACGTtctagaAGAGGTAGCAGCAGCAACAGTG
mSrsf1 CDS forward	(BamHI)-(Kozak)-(Flag)-(forward primer from start codon)	cgcGGATCCgccaccatggattacaaggatgacgacgataagATGTCGGGAGGTGGTGTGAT
mSrsf1 CDS reverse	(XbaI)-(reverse primer from the last codon before stop codon)	ACGTtctagaTGTACGAGAGCGAGATCTGCTAT

Supplementary Table S2: miScript Primer Assays (Qiagen)

microRNA	Species	Catlogue no.
mir-517a-3p	Human	MS00004459
mir-525-5p	Human	MS00004557
miR-10b-5p	Mouse	MS00032249
miR-107-3p	Mouse	MS00032235
miR-122-5p	Mouse	MS00001526
miR-187-3p	Mouse	MS00001750
miR-675-3p	Mouse	MS00006391
miR-1192-5p	Mouse	MS00032263
miR-2b-3p	Drosophila	MS00017983
miR-14-3p	Drosophila	MS00018256
miR-276a-3p	Drosophila	MS00018130
miR-77-3p	C.elegans	MS00020048
miR-230-3p	C.elagans	MS00019383
RNU6B		MS00033740

Supplementary Table S3: Regular RT-PCR Primer Pairs

Gene	Accession no.	F/R	Position	Size	Sequence	Remarks
GAPDH (Human)	NM_002046	Forward	122-141	434bp	GAGTCAACGGATTTGGTCGT	
		Reverse	555-536		GGAGGCATTGCTGATGATCT	
PABPN1 (Human)	NM_004643	Forward	1807-1826	383bp	CGTTGGCAATGTGGACTATG	Same primer pair for mouse Pabpn1
		Reverse	2189-2170		ACCATGATGTCGCTCTAGCC	Same primer pair for mouse Pabpn1
PABPC1 (Human)	NM_002568	Forward	1234-1253	459bp	GCACAGAAAGCTGTGGATGA	
		Reverse	1692-1673		TACAGGGTTGGGAACAGCTC	
PABPC3 (Human)	NM_030979	Forward	1928-1947	459bp	AACAGTGCTACCGGTGTTCC	
		Reverse	2386-2367		CCAAACCATGTTTCCTTGCT	
PABPC4 (Human)	NM_001135654	Forward	1623-1642	458bp	AGGATGCCAATAAGGCTGTG	
		Reverse	2080-2061		GGCAGGAAGTGCTCTCATTC	
PABPC5 (Human)	NM_080832	Forward	703-722	493bp	CATTCCGCCTTATGTGGTCT	
		Reverse	1195-1176		GCAGCCTCGTGTGTCTCATA	

Gene	Accession no.	F/R	Position	Size	Sequence	
Gapdh (Mouse)	NM_008084	Forward	150-169	401bp	CCCTTCATTGACCTCAACTA	
		Reverse	550-531		CCAAAGTTGTCATGGATGAC	
Pabpn1 (Mouse)	NM_019402	Forward	526-545	383bp	CGTTGGCAATGTGGACTATG	Same primer pair for human PABPN1
		Reverse	908-889		ACCATGATGTCGCTCTAGCC	Same primer pair for human PABPN1
Pabpc1 (Mouse)	NM_008774	Forward	1865-1884	425bp	GAGACCAGCTTCCTCACAGG	
		Reverse	2289-2270		CAACCTTTGAGCGGAGAGAC	
Pabpc2 (Mouse)	NM_011033	Forward	1785-1804	500bp	AGGGTCAGGAACCTTGGACT	
		Reverse	2284-2265		TTTCTTTTGGGAGGTTGCTG	
Pabpc4 (Mouse)	NM_130881	Forward	985-1004	464bp	GAAAATCTGGGGTGGGAAAT	
		Reverse	1448-1429		CACAGCCTTATTGGCATCCT	
Pabpc4 (Mouse)	NM_130881	Forward	1914-1933	427bp / 292bp	GCAGCTGGTGGCTATTTTGT	Variant1; 427bp
		Reverse	2340-2321		GGACACTGGAGGCGTACTTG	Variant2; 292bp
Pabpc5 (Mouse)	NM_053114	Forward	1022-1041	436bp	TGCTAGAACTGCATGGCAAG	
		Reverse	1457-1438		GCCCAAGCTATGTAGGCACT	
Pabpc6 (Mouse)	NM_001163836	Forward	1680-1699	481bp	TACACAGCCACATGTCAGCA	
		Reverse	2160-2141		CCTGACTCTGACTTGGCACA	
